# Adaptor protein XB130 regulates the aggressiveness of cholangiocarcinoma

**DOI:** 10.1371/journal.pone.0259075

**Published:** 2021-11-15

**Authors:** Pirawan Poosekeaw, Chawalit Pairojkul, Banchob Sripa, Prakasit Sa Ngiamwibool, Sitthichai Iamsaard, Chadamas Sakonsinsiri, Raynoo Thanan, Piti Ungarreevittaya

**Affiliations:** 1 Department of Pathology, Faculty of Medicine, Khon Kaen University, Khon Kaen, Thailand; 2 Department of Anatomy, Faculty of Medicine, Khon Kaen University, Khon Kaen, Thailand; 3 Department of Biochemistry, Faculty of Medicine, Khon Kaen University, Khon Kaen, Thailand; 4 Cholangiocarcinoma Research Institute, Khon Kaen University, Khon Kaen, Thailand; University of Campinas, BRAZIL

## Abstract

Cholangiocarcinoma (CCA) is a group of heterogenous malignancies arising from bile duct epithelium with distinct pathological features. Adaptor proteins have implicated in cell proliferation, migration, and invasion of different cancer cells. The objective of this study was to assess whether the adaptor protein XB130 (AFAP1L2) is a critical biological determinant of CCA outcome. XB130 expression levels were investigated in four CCA cell lines compared to an immortalized cholangiocyte cell line by Western blotting. Small interfering (si) RNA-mediated *XB130* gene silencing was conducted to evaluate the effects of reduced XB130 expression on cell proliferation, migration, and invasion by MTT, transwell migration and cell invasion assay. The immunohistochemical quantification of XB130 levels were performed in surgically resected formalin-fixed, paraffin-embedded specimens obtained from 151 CCA patients. The relationship between XB130 expression and the clinicopathological parameters of CCA patients were analyzed. Our results showed that XB130 was highly expressed in KKU-213A cell line. Knockdown of XB130 using siRNA significantly decreased the proliferation, migration, and invasion properties of KKU-213A cells through the inhibition of PI3K/Akt pathway, suggesting that XB130 plays an important role in CCA progression. Moreover, elevated XB130 expression levels were positive relationship with lymphovascular space invasion (LVSI), intrahepatic type of CCA, high TNM staging (stage III, IV), high T classification (T3, T4), and lymph node metastasis. We provide the first evidence that the overexpression of XB130 is associated with tumorigenic properties of CCA cells, leading to CCA progression with aggressive clinical outcomes.

## Introduction

Cholangiocarcinoma (CCA) is a malignancy of biliary epithelial cells and its incidence has increased worldwide [1]. The risk factors of CCA are heterogeneous and differ globally [1]. Primary sclerosing cholangitis is the most typical risk factor for CCA in the Western countries, while in several Asian countries *Opisthorchis viverrini* is determined as the key risk factor for CCA [2]. CCA is classified into intrahepatic and extrahepatic types, based on its anatomical location along the biliary tree. Intrahepatic CCA develops within the small and second order large bile duct branches, which are situated in the liver parenchyma. Extrahepatic CCA involves the extrahepatic large bile duct location within biliary tree and the hepatoduodenal ligament [3]. CCA is histologically graded into well, moderately, and poorly differentiated adenocarcinomas [4]. CCA remains a major health problem which leads to high mortality in Thailand, notably in the Northeastern region [5]. Because of a high degree of malignancy, rapid progression, low surgical resection rates and high recurrence rates, CCA prognoses are poor.

Several independent prognostic factors for CCA have been identified, including serum carcinoembryonic antigen (CEA) levels, alkaline phosphatase levels, relapse of disease, intraoperative blood transfusion, lymph node metastasis, TNM staging [6, 7], histological differentiation of tumors, surgical margins, types of CCA, Caudal Type Homeobox 2 (CDX2), and human epidermal growth factor receptor 2 (*HER2)* gene amplification [8]. It has been shown that serum CEA levels of greater than 2.5 ng/mL are considered as an independent poor prognostic factor for resectable CCA patients [6, 7]. CCA patients with CDX2-positive tumors were reported to have significantly better survival than those with CDX2-negative tumors [9]. Moreover, the overexpression of *HER-2* gene amplification using chromogenic *in situ* hybridization (CISH) is an independent prognostic factor for survival in the subgroup of extrahepatic CCA patients with lymph node metastases [7]. However, the estimated overall 5-year survival rate of CCA is less than 10% and only approximately one third of the CCA patients are possible for receiving curative treatment at the diagnostic time [10]. Therefore, the search for novel prognostic marker is still needed for improvement of CCA therapy.

In the development of the human embryo, the liver, extrahepatic biliary ducts, gallbladder, and pancreas duct arise from the same epithelial anlage [11]. Accumulating evidence suggests that CCA and pancreatic ductal adenocarcinoma have similar pathology, background, and development [12]. Zhang et al. demonstrated that the high expression of XB130 is an independent prognostic marker to predict poor outcome after surgical resection of pancreatic ductal adenocarcinoma [13]. However, the roles and expression levels of XB130 protein in CCA, which is a malignancy developing from the biliary epithelial cells, have never been investigated.

XB130, which is also known as actin filament-associated protein 1 like 2 (AFAP1L2), is an important member of the actin filament-associated protein (AFAP) family of adaptor proteins. *XB130* gene is located on human chromosome 10q25.3 and encodes a protein of 818 amino acids [14]. XB130 is an adaptor protein involved in cell proliferation, survival, and migration [15–17]. This adaptor protein has been shown to cause the activation of kinases and related downstream proteins in many signaling pathways [18]. It can also promote interactions between protein binding partners, triggering signaling cascades. For instance, XB130 binds to the p85α subunit of phosphatidylinositol-3-kinase (PI3K) and activates protein kinase B (Akt). The excessive activation of PI3K/Akt signaling pathway promotes cell proliferation by regulating cell cycle during G1 to S phase progression and by inducing cell migration and tumor cell survival [19]. XB130 is mainly distributed in the cytosol, where the chemical reactions predominantly occur and is associated with physiological processes and oncogenesis of certain malignancies [18]. Functional roles of XB130 have been examined in various types of cancer both *in vitro* and *in vivo*. For example, the overexpression of XB130 in human esophageal squamous cell carcinoma (ESCC) has shown to be associated with cell cycle progression and poor prognosis [20]. The previous studies in lung and thyroid cancer cells demonstrated that XB130 is a potential regulator of tyrosine kinase-mediated signaling and substrate that control of cell proliferation and apoptosis [19, 21]. Knockdown of XB130 in human lung and thyroid cancer cells by small interfering RNA (siRNA) suppressed cell cycle progression and induced spontaneous apoptosis [19, 21]. However, little is known about the roles of XB130 in CCA.

In this study, we aimed to investigate: i) the expression levels of XB130 in four different CCA cell lines (KKU-100, KKU-213A, KKU-213C, and KKU-023) compared to an immortalized cholangiocyte cell line (MMNK1); ii) the effects of decreased XB130 expression on cell proliferation, cell migration and invasion in XB130-expressing KKU-213A cells; iii) the expression levels of XB130 in CCA tissues using immunohistochemistry; and iv) the relationship between the XB130 expression levels with the clinicopathological features and survival rates of CCA patients.

## Materials and methods

### Cell culture

For different CCA cell lines (KKU-100, KKU-213A, KKU-213C, and KKU-023) and an immortal cholangiocyte cell line (MMNK1) were obtained from the Cholangiocarcinoma Research Institute, Khon Kaen University, Thailand. KKU-213A and KKU-213C CCA cell lines were established and characterized by Sripa B [22]. All cell lines were cultured in Ham′s F-12 (Life technologies, Grand Island, NY, USA) containing 10% heat-inactivated fetal bovine serum, 100 U/mL penicillin and 100 ug/mL streptomycin (Life technologies, Grand Island, NY, USA). Cells were incubated at 37°C in a humidified incubator with an atmosphere of 5% CO_2_.

### Screening of the XB130 expression in cell lines using Western blot analysis

Cells were lysed on ice in RIPA buffer (150 mM NaCl, 50 mM Tris-HCl, 1% (v/v) Triton X-100, 1% (w/v) sodium deoxycholate, 0.1% (w/v) sodium dodecyl sulfate (SDS) supplemented with protease inhibitor cocktail (Thermo Fisher Scientific, MA, USA; S1 Appendix) The protein concentration was measured using Pierce BCA Protein assay kit (Pierce Biotechnology, Rockford, USA). Proteins in the total cell lysate were separated onto SDS-PAGE (4% stacking gel and 8% separating gel) and transferred onto a polyvinylidene difluoride (PVDF) membrane (Bio-Rad, CA, USA). After blocking the blot in a solution of 5% skimmed milk in Tris-buffered saline (TBS) pH 8.0 at room temperature for 1 h, membrane-bound proteins were probed with mouse anti-XB130 monoclonal antibody (1:1000; Santa Cruz, USA), rabbit anti-E-cadherin antibody (1:1000, Cell Signaling Technology, Danvers, MA, USA), mouse anti-vimentin antibody (1:1000, Agilent Dako, Santa Clara, CA, US), rabbit anti-Akt antibody (1:1000, Cell Signaling Technology, Danvers, MA, USA), rabbit anti-Ser473 phosphorylated Akt (pAkt) antibody (1:1000, Cell Signaling Technology, Danvers, MA, USA) and mouse anti-β-actin antibody (1:60000, Sigma, Louis, MO, USA) overnight at 4°C. The membrane was washed and incubated with peroxidase-labelled anti-rabbit or anti-mouse secondary antibodies (1:2000) for 1 h at room temperature. Finally, immunoreactive bands were visible to Amersham^™^ ECL^™^ Prime Western Blotting Detection Reagent (GE Healthcare, Buckinghamshire, UK) for chemiluminescence detection which was performed using an Amersham Imager^™^ 600 (GE Healthcare Bio-Sciences AB, Uppsala, Sweden). Original images of Western blot results are shown in S1–S7 Figs.

### Knockdown of XB130 in an CCA cell line using siRNA

Specific siRNA against XB130 (siXB130), ON-TARGET plus Human XB130 siRNAs; cat. no. L-014917-02-0005, and a negative control or scramble, ON-TARGET plus Non-targeting Pool siRNAs; cat no. D-001810-10-05, were purchased from GE Healthcare Dharmacon Inc. (Lafayette, CO, USA). KKU-213A cells (12 x 10^4^ cells/well) were transfected with 50 nM of siXB130 or scramble using lipofectamine^®^ RNAiMax (Invitrogen, Carlsbad, CA, USA) according to the manufacturer’s protocol. After 48 h transfection, cells were used for detection of XB130 expression by western blot analysis as well as cell proliferation, invasion and migration assays.

### Cell proliferation assay

At 48 h after XB130 knockdown, treatment with scramble and siXB130, the cells were trypsinized and seeded onto 96-well plates (3.0 × 10^4^ cells) in quintuplicate. Cell proliferation was measured using the methyl thiazolyl tetrazolium (MTT) assay as previously described [23].

### Cell invasion assay

Cell invasion assay was performed using a 24-well Matrigel Invasion Chamber (Discovery Labware, Inc, Bedford, MA, USA) as previously described [24]. The cell invasion was determined at 24 h after incubation.

### Cell migration assay

The migration ability of CCA cells across the membrane filter of 8 μm pore-size was measured using a Boyden chamber in a 24-well plate (Corning, NY, USA) as previously described [23]. The number of cells migrated across the membrane was determined at 24 h.

### Human cholangiocarcinoma tissues

The formalin-fixed and paraffin-embedded CCA tissues used in this study were the leftover tissues after pathological diagnosis of CCA (n = 151 cases) in the Srinagarind Hospital, Khon Kaen University, from January 2007 to August 2017 and performed tissue microarray (4 cores, 2 mm). The protocol of this study was approved by the Ethics Committee for Human Research, Khon Kaen University (HE621409).

### Immunohistochemical staining

The immunohistochemistry (IHC) method for XB130 protein was performed on tissue microarray slide sections, using a VENTANA BenchMark XT Slide Staining System (Roche Diagnostics, NJ, USA). The tissue microarray slides were cut at 4 μm thickness and dried in a hot-air oven overnight at 50°C. Immunohistochemical staining was carried out following the manufacturer’s recommended protocol. Mouse anti-AFAP1L2/XB130 monoclonal antibody (1:100; Santa Cruz, TX, USA) was used as a primary antibody and its presence was visualized using an OptiView DAB IHC detection kit (Ventana, AZ, USA). For XB130 assessment, the stained tissue microarray slides were entirely scanned to assign the scores using a ScanScope^®^ XT digital slide scanner (Leica Biosystems, Singapore).

### Evaluation of immunohistochemical staining

The semi-quantitative analysis of IHC scores using an ImageJ software, Java-based image processing program developed at the National Institutes of Health and the Laboratory for Optical and Computational Instrumentation, University of Wisconsin [25, 26]. The quantification methods were performed following the previous report [27]. XB130 expression levels (IHC scores) of 151 CCA tissues examined ranged from 28.00 to 220.45.

### Clinicopathological parameters of CCA tissues

The clinicopathologic variables included age, gender, type of CCA, histological type, histologic differentiation, LVSI, T classification, N classification (lymph node metastasis), M classification, TNM staging, and surgical margins were used for data analysis. By anatomical location of the tumor, CCA was classified into intrahepatic CCA and extrahepatic CCA. Histologic differentiation of CCA was classified by histology into well, moderately, and poorly differentiated adenocarcinomas [4]. Histologic type was defined as papillary or tubular base on architectural growth pattern. The LVSI is the histologic visible invasion of a cancer cells into lymphatic and/or blood vessels which is the cause of lymphatic and vascular spreading. Lymph node metastasis is cancer that has spread to lymph nodes and a surgical margin is the margin of apparently non-tumorous tissue around a tumor that has been surgically removed [28]. The TNM classification of Malignant Tumors seventh edition is an anatomically based system that records the primary size, regional nodal extent of the tumor, and distance metastasis. TNM is a notation system that describes the stage of a cancer, T category describes the primary tumor site, N category describes the regional lymph node involvement and M category describes the presence or otherwise of distant metastatic spread [29].

### Statistical analyses

The relationship between XB130 expression levels with clinicopathological features were performed by linear regression. Survival analysis was performed using the Kaplan-Meier method and the difference between survival curves was calculated using the log-rank test. Cox’s proportional hazards models of univariate and multivariate analyses were performed to define risks and identify independent prognostic factors of XB130 protein expression. Univariate and multivariate logistic regression analyses were performed to identify factors affecting lymph node metastasis (N1) in CCA. All statistical analyses were performed using STATA 10.1 software (StataCorp LLC, TX, USA). Statistical significance is defined as a *P*-value < 0.05.

## Results

### Evaluation of XB130 expression levels in CCA cell lines and an immortal cholangiocyte cell line

**Fig 1:**
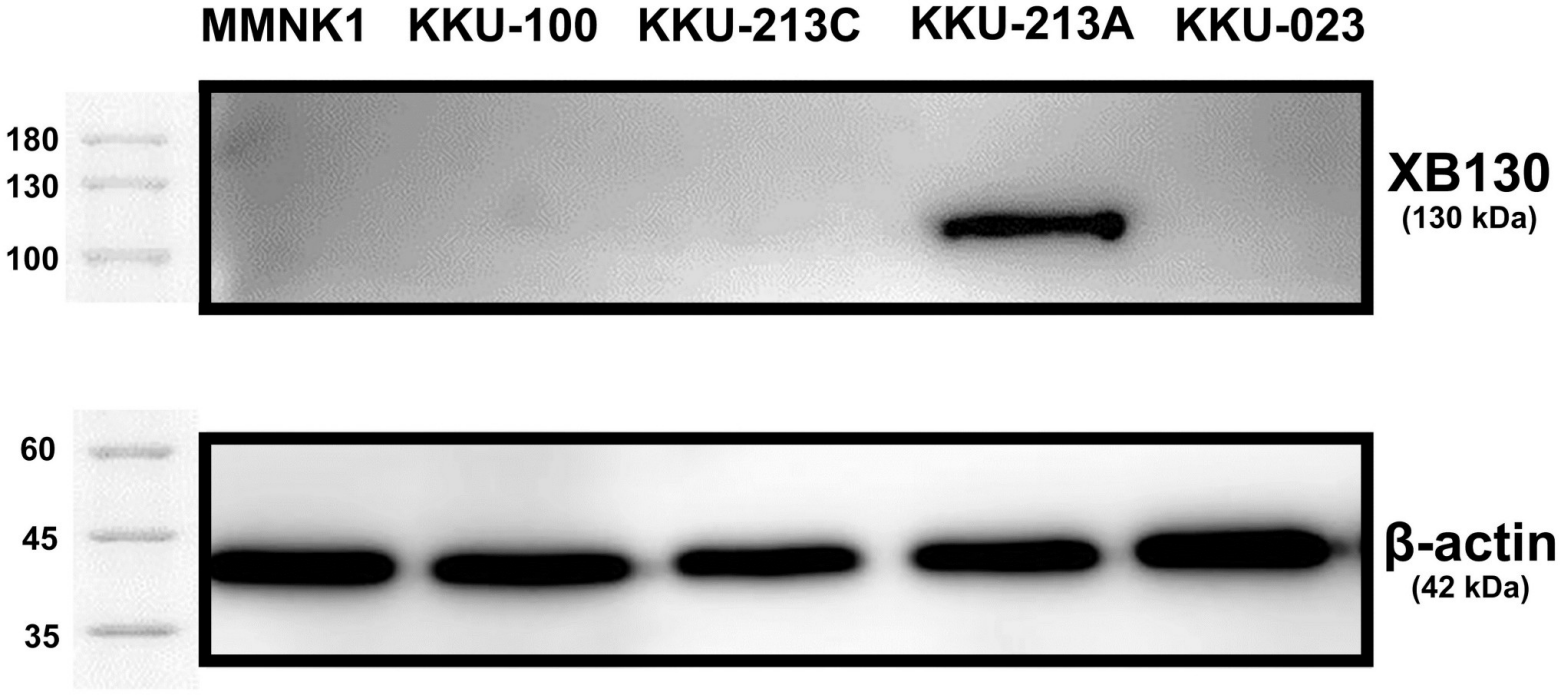
Western blot analysis of XB130 in MMNK1, KKU-100, KKU-213C, KKU-213A and KKU-023 cell lines, with β-actin as a loading control.

XB130 expression levels in the immortal cholangiocyte (MMNK1) and four CCA cell lines (KKU-100, KKU-213C, KKU-213A, and KKU-023) were measured semi-quantitatively using Western blot analysis. The results showed that XB130 was not expressed in MMNK1 cells (Fig 1). Among four studied CCA cell lines, XB130 was highly expressed in the KKU-213A cell line, but it was not expressed in other cells lines (*i*.*e*., KKU-100, KKU-213C and KKU-023 cell lines).

### Roles of XB130 in proliferation, migration and invasion in CCA

**Fig 2:**
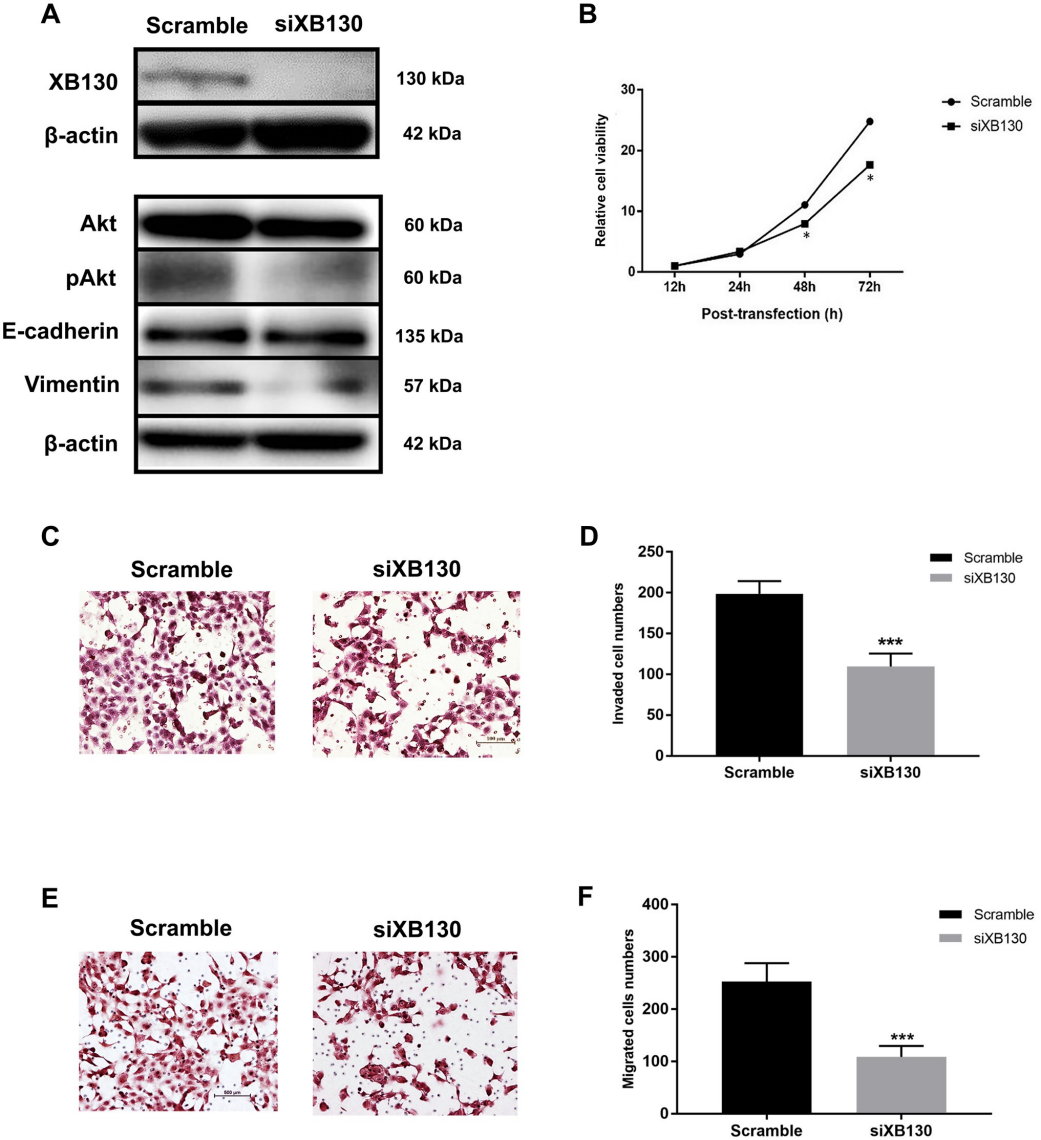
Effects of siXB130 on proliferation, motility and invasiveness of KKU-213A cell line. A): The expressions of XB130 (130 kDa), E-cadherin (~135 kDa), vimentin (~54 kDa), Akt (~60 kDa), pAkt (~60 kDa) in siXB130 and scramble groups measured by western blotting, with β-actin as a loading control. B): MTT assays of the scramble and siXB130 treated cells. C): Hematoxylin-staining invasive cells from cell invasion assays of the scramble and siXB130 treated cells. D): Graphical represents numbers of invasive cells from cell invasion assays. E): Hematoxylin staining migrated cells from cell migration assays. F): Graphical represents numbers of migrating cells from cell migration assays. The asterisk (*) indicates statistical significance at *P*<0.05 and asterisks (***) indicates statistical significance at *P* < 0.001.

The effects of down-regulation of XB130 by siRNA on cell proliferation, migration and invasion were examined in KKU-213A cell line. *XB130* gene silencing using siRNA technology successfully suppressed the expression levels of XB130 compared with the scrambled control at 48 h after treatment (Fig 2A). The cell proliferation rates and cell invasion and migration activities of siXB130-treated KKU-213A cell line were significantly reduced compared to control cells (Fig 2B–2F).

### XB130 activates the PI3K/Akt pathway and alters metastasis-associated proteins in CCA

To explore the mechanisms underlying XB130 expression, the possible downstream targets of XB130 including PI3K downstream (Akt), Ser473 phosphorylated Akt (pAkt) and epithelial-mesenchymal transition (EMT) markers (E-cadherin and vimentin) were measured in the XB130 knockdown-CCA cell lines using western blot analysis. The results indicated that XB130 siRNA effectively reduced XB130 protein levels; a phenomenon associated with decreased the expression and phosphorylation of Akt as well as the expression of EMT inducing protein (vimentin) as shown in Fig 2A. Our findings suggest that XB130 has an oncogenic property that can induce cell proliferation, migration and invasion in CCA via the activation of PI3K/Akt pathway leading to increasing of EMT processes.

### Expression levels of XB130 in CCA tissues

**Fig 3:**
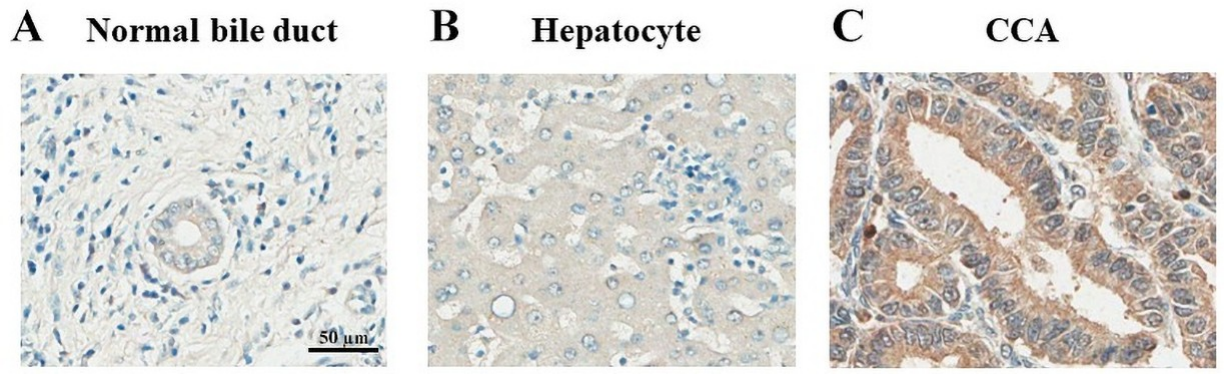
XB130 expression patterns in: A) normal bile duct; intensity = 48, B) hepatocyte; intensity = 72 and C) CCA intensity = 220 analyzed by IHC. The scale bar equals 50 μm.

The expression levels of XB130 protein in CCA tissues (n = 151) were determined using immunohistochemistry. The protein was highly localized in cytoplasm of CCA cells, whereas it was weakly stained in normal bile duct and hepatocyte cells. The representative results of normal bile duct, hepatocyte and CCA tissues with XB130 expression levels were shown in Fig 3.

### Relationship between the XB130 expression levels and clinicopathological data of CCA patients

The relationship between XB130 protein expression levels and the clinicopathological features of CCA patients were summarized in Table 1. No relationship was observed between XB130 expression and the following variables: age (*β* = -0.0023, *P* = 0.976), gender (*β* = 0.0042, *P* = 0.958), histological type (*β* = 0.0262, *P* = 0.749), margin status (*β* = -0.1305, *P* = 0.110), moderate differentiation (*β* = 0.1238, *P* = 0.128), poor differentiation (*β* = 0.1477, *P* = 0.070) or M classification (*β* = 0.074, *P* = 0.365). The XB130 expression was significantly positive relationship with LVSI (*β* = 0.2274, *P* = 0.005), intrahepatic type of CCA (*β* = 0.1623, *P* = 0.046), stage III of TNM staging (*β* = 0.4048, *P* < 0.001), stage IV of TNM staging (*β* = 0.2363, *P* = 0.012), T3 classification (*β* = 0.4314, *P* < 0.001), T4 classification (*β* = 0.3327, *P* < 0.001) and lymph node metastasis (*β* = 0.2060, *P* = 0.011).

**Table 1:** Relationship between XB130 expression and clinicopathologic features of CCA patients.

Variable	XB130 expression			
	*Coef*.	*95% CI*	*β*	*P*-value
**Gender**				
Female/male	-0.12	-8.9111–8.6517	0.0042	0.976
**Age**				
Years	0.41	-15.4027–16.2285	-0.0023	0.958
**LVSI**				
Yes/No	22.08	6.7806–37.3836	0.2274	0.005
**Histological type**				
Tubular/Papillary type	2.48	-12.8509–17.8225	0.0262	0.749
**Margin status**				
Yes/No	-12.75	-28.4395–2.9211	-0.1305	0.110
**Type of CCA**				
Intrahepatic/Extrahepatic	17.03	0.2762–33.8017	0.1623	0.046
**Histologic Differentiation**				
Well		1		
Moderated	15.78	-4.5935–36.1560	0.1238	0.128
Poor	50.16	-4.1080–104.4313	0.1477	0.070
**TNM staging**				
I		1		
II	10.58	-10.0906–31.2698	0.0970	0.313
III	41.17	21.5696–60.7782	0.4048	< 0.001
IV	28.41	6.2269–50.6001	0.2363	0.012
**T classification**				
T1		1		
T2	3.93	-11.8204–19.6827	0.0412	0.623
T3	56.87	35.8058–77.9391	0.4314	< 0.001
T4	54.32	29.1228–79.5356	0.3327	< 0.001
**N classification**				
N1/N0	19.71	4.5583–34.8619	0.2060	0.011
**M classification**				
M1/M0	8.91	-10.494–28.3251	0.074	0.365

*Definition of TNM staging system described in the S1 Data.

*Coef, coefficients; *95% CI*, 95%confidence interval; *β*, correlation coefficient.

### Univariate and multivariate analyses of clinicopathological variables influencing survival and lymph node metastasis in CCA

Clinicopathological parameters of 151 CCA patients (95 males and 56 females) with an age range of 34–79 years were analyzed. The impact of 12 variables, including age, gender, type of CCA, histological type, histologic differentiation, LVSI, T classification, N classification (lymph node metastasis), M classification, TNM staging, surgical margins and XB130 expression, on survival was investigated using univariate and multivariate analyses. On univariate analysis using the Cox’s proportional hazards model, the poor survival was associated with the following parameters: LVSI (HR = 1.6150, *P* = 0.007), tubular histological type (HR = 1.6348, *P* = 0.005), positive margin status (HR = 1.6777, *P* = 0.003), TNM staging (stage IV, HR = 4.2664, *P* ≤ 0.001), T classification (T4, HR = 2.0254, *P* = 0.028), N1 classification (HR = 2.3754, *P* ≤ 0.001) and M1 classification (HR = 3.4339, *P* ≤ 0.001), but not with gender (HR = 1.0722, *P* = 0.659), age (HR = 1.0139, *P* = 0.163), intrahepatic type of CCA (HR = 1.1532, *P* = 0.451), moderate differentiation, (HR = 1.5331, *P* = 0.055), poor differentiation, (HR = 0.4662, *P* = 0.287), T classification (T2, HR = 1.3788, *P* = 0.111), T classification (T3, HR = 1.1805, *P* = 0.543), TNM staging (stage II, HR = 1.2338, *P* = 0.400), TNM staging (stage III, HR = 1.4768, *P* = 0.101), XB130 expression (HR = 1.000, *P* = 0.356) (Table 2). The Kaplan-Meier survival curves showed the comparison of the survival rate in 151 cases of CCA patients between gender, histological type, type of CCA, M classification, LVSI, margin status, histologic differentiation, TNM staging, T classification and N classification. Tubular histological type (*P* = 0.0052), M1 classification (*P* < 0.0001), LVSI (*P* = 0.0062), positive margin status (*P* = 0.0041), stage IV of TNM staging (*P* < 0.001) and N1 classification (*P* < 0.001) were significant related to prognosis as shown in S8 Fig.

**Table 2:** Univariate analysis (Cox’s proportional hazards model) for overall survival of CCA patients.

Characteristics	Univariate		
	*HR*	*95% CI*	*P*-value
**Gender**			
Female/male	1.0722	0.7569–1.5187	0.659
**Age**			
Years	1.0139	0.9943–1.0339	0.163
**LVSI**			
Yes/No	1.6150	1.1404–2.2870	0.007
**Histological type**			
Tubular /Papillary type	1.6348	1.1583–2.3073	0.005
**XB130 expression**			
Intensity	1.0000	0.9980–1.0053	0.356
**Margin status**			
Yes/No	1.6777	1.1873–2.3708	0.003
**Type of CCA**			
Intrahepatic/Extrahepatic	1.1534	0.7924–1.6788	0.451
**Histologic Differentiation**			
Well		1	
Moderated	1.5331	0.9907–2.3722	0.055
Poor	0.4662	0.1144–1.8988	0.287
**TNM staging**			
I		1	
II	1.2338	0.7559–2.0137	0.400
III	1.4768	0.9266–2.3538	0.101
IV	4.2664	2.4784–7.3443	< 0.001
**T classification**			
T1		1	
T2	1.3788	0.9291–2.0461	0.111
T3	1.1805	0.6919–2.0140	0.543
T4	2.0254	1.0781–3.8048	0.028
**N classification**			
N1/N0	2.3754	1.6709–3.3769	< 0.001
**M classification**			
M1/M0	3.43396	2.1942–5.3741	< 0.001

In addition, multivariate analysis using Cox’s proportional hazards model showed that only N1 classification (HR = 1.8707, *P* = 0.002) and M1 classification (HR = 2.3925, *P* < 0.001) were significantly correlated with poor survival. The two clinicopathologic variables, *i*.*e*., N and M classifications, can be used as independent prognostic factors (Table 3). Moreover, the results obtained from multivariate analysis using logistic regression demonstrated that M1 and LVSI were independent factors for lymph node metastasis (Table 4).

**Table 3:** Multivariate analysis (Cox’s proportional hazards model) for overall survival of CCA patients.

Characteristics	Multivariate		
	*HR*	*95% CI*	*P*-value
**N classification**			
N1/N0	1.8707	1.2589–2.7797	0.002
**M classification**			
M1/M0	2.3925	1.4589–3.9237	0.001

**Table 4:** Univariate and multivariate analyses (logistic regression model) of factor correlated with lymph node metastasis (N1).

Clinicopathologic characteristics	Univariate			Multivariate		
	Odds ratio	95% CI	*P*-value	Odds ratio	95% CI	*P*-value
**M category (+)**	29.84	6.7488–131.9561	< 0.001	22.17	4.9082–100.1789	< 0.001
**LVSI (+)**	5.32	2.4970–11.3455	< 0.001	3.69	1.6187–8.4202	0.002

## Discussion

The main finding of this study was that XB130 overexpression in CCA was related to cell proliferation, migration, and invasion, leading to CCA progression with aggressive clinical outcomes. These results suggest that the expression levels of XB130 in CCA cell lines, XB130 was highly expressed in the KKU-213A cell line, but it was not expressed in other cells lines (*i*.*e*., KKU-100, KKU-213C and KKU-023 cell lines). The expression levels of XB130 in CCA cell lines may not be involved in the transformation from cholangiocytes to CCA cells. According to the cell line’s properties, KKU-213A has the highest proliferation, invasion and migration rates among the other tested cell lines [22, 30]. Thus, in CCA, XB130 may play a critical role in tumor cell progression, but not in tumorigenesis. The KKU-213A cell line was selected for further functional analysis of XB130 using specific siRNA technique. Significant inhibition of cell proliferation, migration, and invasion caused by *XB130* silencing in KKU-213A cells suggests that XB130 plays an important role in the progression of CCA by promoting cell growth, migration, and invasion.

These findings are consistent with previous studies on XB130 in a variety of cancers, *e*.*g*., thyroid tumor [19, 31], prostate cancer [15], lung cancer [17], gastric cancer [32], breast cancer [33], colorectal cancer [34], hepatocellular carcinoma [35], and skin tumor [24]. XB130 has been suggested to play significant roles in cancer progression through PI3K/Akt signaling pathway, leading to an increase in tumorigenic properties, including cell growth, migration, invasion and epithelial–mesenchymal transition (EMT) process [17, 31, 35–37]. It is worth noting that in CCA, the PI3K/Akt signaling pathway was constitutively activated and was involved in cell proliferation, migration and invasion [38, 39]. Suppression of the PI3K/Akt signaling pathway by various anti-cancer drugs could also inhibit CCA cell progression [35, 40–43]. From the above mentioned, we hypothesized that XB130 activates PI3K/Akt pathway resulting in the phosphorylation of Akt (pAkt) and induction of EMT process in CCA cells. To prove the hypothesis, the detections of PI3K/Akt pathway-downstream marker (pAkt) and EMT markers (E-cadherin and vimentin) expression levels in siXB130 and the vertical control cells were measured using western blot analysis. The results showed that pAkt and vimentin expression levels were decrease in the XB130 knockdown-CCA cell line. Therefore, we concluded that the overexpression of XB130 in CCA accelerates the cancer progression via PI3K/Akt signaling pathway.

The XB130 expression levels in clinical tissues were further analyzed to assess its potential for being used as an accurate prediction of survival of patients with CCA and as a valid biological indicator for the aggressiveness of CCA. The XB130 expression level was not an independent prognosis factor for survival in CCA. It should be noted that distant metastasis (M1-classification) has more potential clinical use to predict for the risk of lymph node metastasis in CCA than the LVSI. The XB130-mediated aggressiveness of CCA was confirmed by the biological function assays, *i*.*e*., cell proliferation, invasion, and migration in cell lines. In CCA tissues, the elevated XB130 expression levels had positive relationship with lymphovascular space invasion (LVSI), intrahepatic type of CCA, high TNM staging (stage III, IV), high T classification (T3, T4), and N1 classification (lymph node metastasis). The high T classification, LVSI, lymph node metastasis, and high TNM staging all indicate tumor extension and invasion, which supported the *in vitro* investigation of cell proliferation and invasion in KKU-213A cells. The Kaplan-Meier survival curves showing LVSI, margin status, TNM staging, and N classification were significantly related to prognosis. Although, XB130 level was not directly associated with survival outcome in CCA patients, but it was associated with other factors correlated with survival, including LVSI, high TNM staging and lymph node metastasis. Therefore, our findings demonstrated that the adapter protein XB130 is a significant player in CCA progression and metastasis.

## Conclusion

In this work, the expression of XB130 was assessed along with its impact on CCA proliferation, migration, and invasion *in vitro*. Furthermore, high expression levels of XB130 in tissues reflect a role in predicting the aggressive behavior of CCA, as evidenced by significant risk factors, i.e., LVSI, lymph node metastasis, and high TNM staging in the patients. Targeting XB130 may assist in slowing tumor growth and decreasing cell migration and invasion. Therefore, high XB130 expression might be considered as an indicator of aggressive CCA and a potential target for CCA therapy.

## Supporting information

S1 Fig Original image of western blot result of XB130 expression in cell lines. (PDF)

S2 Fig Original image of western blot result of β-actin expression in cell lines. (PDF)

S3 Fig Original image of western blot result of phosphorylation of Akt expression in media, scramble and siXB130 cells. (PDF)

S4 Fig Original image of western blot result of Akt expression in media, scramble and siXB130 cells. (PDF)

S5 Fig Original image of western blot result of E-cadherin expression in media, scramble and siXB130 cells. (PDF)

S6 Fig Original image of western blot result of vimentin expression in media, scramble and siXB130 cells. (PDF)

S7 Fig Original image of western blot result of β-actin in media, scramble and siXB130 cells. (PDF)

S8 Fig The Kaplan-Meier analysis of clinical data. (PDF)

S1 Data Definition of TNM staging system. (PDF)

S2 Data Evaluation of immunohistochemical staining by ImageJ. (PDF)

S1 Appendix (PDF)

## References

[1] Al-Bahrani R, Abuetabh Y, Zeitouni N, Sergi C. Cholangiocarcinoma: risk factors, environmental influences and oncogenesis. Ann Clin Lab. 2013; 43(2):195–210. Epub 2013/05/23. . 23694797

[2] Sriamporn S, Pisani P, Pipitgool V, Suwanrungruang K, Kamsa‐ard S, Parkin D. Prevalence of Opisthorchis viverrini infection and incidence of cholangiocarcinoma in Khon Kaen, Northeast Thailand. Trop Med Int Health. 2004; 9(5):588–94. doi: 10.1111/j.1365-3156.2004.01234.x . 15117303

[3] Klatskin G. Adenocarcinoma of the hepatic duct at its bifurcation within the porta hepatis: An unusual tumor with distinctive clinical and pathological features. Am J Med. 1965; 38(2):241–56. doi: 10.1016/0002-9343(65)90178-6 . 14256720

[4] Nakanuma Y, Sato Y, Harada K, Sasaki M, Xu J, Ikeda H. Pathological classification of intrahepatic cholangiocarcinoma based on a new concept. World J Hepatol. 2010; 2(12):419–27. 10.4254/wjh.v2.i12.419 21191517 PMC3010511

[5] Sripa B, Pairojkul C. Cholangiocarcinoma: lessons from Thailand. Curr Opin Gastroenterol. 2008; 24(3):349–56. doi: 10.1097/MOG.0b013e3282fbf9b3 18408464 PMC4130346

[6] Wirasorn K, Ngamprasertchai T, Chindaprasirt J, Sookprasert A, Khantikaew N, Pakkhem A, et al. Prognostic factors in resectable cholangiocarcinoma patients: Carcinoembryonic antigen, lymph node, surgical margin and chemotherapy. World J Gastrointest Oncol. 2013; 5(4):81–7. Epub 2013/05/15. doi: 10.4251/wjgo.v5.i4.81 23671735 PMC3648667

[7] Kim HJ, Yoo TW, Park DI, Park JH, Cho YK, Sohn CI, et al. Gene amplification and protein overexpression of HER-2/neu in human extrahepatic cholangiocarcinoma as detected bychromogenic in situ hybridization and immunohistochemistry: its prognostic implication in node-positive patients. Ann Oncol. 2007; 18(5):892–7. Epub 2007/02/27. doi: 10.1093/annonc/mdm006 . 17322545

[8] Mao ZY, Guo XC, Su D, Wang LJ, Zhang TT, Bai L. Prognostic Factors of Cholangiocarcinoma After Surgical Resection: A Retrospective Study of 293 Patients. Med Sci Monit. 2015; 21:2375–81. Epub 2015/08/14. doi: 10.12659/MSM.893586 26269932 PMC4540057

[9] Tang H, Wang Z, Lv W, Meng X. The expression and clinicopathological role of CDX2 in intrahepatic cholangiocarcinoma. Intractable Rare Dis Res. 2018; 8(7):106–11. doi: 10.5582/irdr.2018.01047 29862152 PMC5982617

[10] Doherty B, Nambudiri VE, Palmer WC. Update on the diagnosis and treatment of cholangiocarcinoma. Curr Gastroenterol Rep. 2017; 19(1):2. doi: 10.1007/s11894-017-0542-4 . 28110453

[11] Ando H. Embryology of the biliary tract. Dig Surg. 2010; 27(2):87–9. Epub 2010/05/10. doi: 10.1159/000286463 . 20551648

[12] Nakanuma Y, Sato Y. Hilar cholangiocarcinoma is pathologically similar to pancreatic duct adenocarcinoma: suggestions of similar background and development. J Hepatobiliary Pancreat Sci. 2014; 21(7):441–7. Epub 2014/01/21. doi: 10.1002/jhbp.70 . 24446421

[13] Zhang J, Jiang X, Zhang J. Prognostic significance of XB130 expression in surgically resected pancreatic ductal adenocarcinoma. World J Surg Oncol. 2014; 12(1):49. doi: 10.1186/1477-7819-12-49 24581082 PMC3996025

[14] Xu J, Bai XH, Lodyga M, Han B, Xiao H, Keshavjee S, et al. XB130, a novel adaptor protein for signal transduction. J Biol Chem. 2007; 282(22):16401–12. Epub 2007/04/07. doi: 10.1074/jbc.M701684200 . 17412687

[15] Chen B, Liao M, Wei Q, Liu F, Zeng Q, Wang W, et al. XB130 is overexpressed in prostate cancer and involved in cell growth and invasion. Oncotarget. 2016; 7(37):59377–87. Epub 2016/08/11. doi: 10.18632/oncotarget.11074 27509056 PMC5312318

[16] Takeshita H, Shiozaki A, Bai XH, Iitaka D, Kim H, Yang BB, et al. XB130, a new adaptor protein, regulates expression of tumor suppressive microRNAs in cancer cells. PLoS One. 2013; 8(3):e59057. Epub 2013/03/26. doi: 10.1371/journal.pone.0059057 23527086 PMC3602428

[17] Shiozaki A, Shen-Tu G, Bai X, Iitaka D, De Falco V, Santoro M, et al. XB130 mediates cancer cell proliferation and survival through multiple signaling events downstream of Akt. PLoS One. 2012; 7(8):e43646. Epub 2012/08/29. doi: 10.1371/journal.pone.0043646 22928011 PMC3426539

[18] Bai XH, Cho HR, Moodley S, Liu M. XB130-A Novel Adaptor Protein: Gene, Function, and Roles in Tumorigenesis. Scientifica (Cairo). 2014; 2014:903014. Epub 2014/07/06. doi: 10.1155/2014/903014 24995146 PMC4068053

[19] Shiozaki A, Liu M. Roles of XB130, a novel adaptor protein, in cancer. J Clin Bioinforma. 2011; 1(1):10. Epub 2011/09/03. doi: 10.1186/2043-9113-1-10 21884627 PMC3164603

[20] Shiozaki A, Kosuga T, Ichikawa D, Komatsu S, Fujiwara H, Okamoto K, et al. XB130 as an independent prognostic factor in human esophageal squamous cell carcinoma. Ann Surg Oncol. 2013; 20(9):3140–50. Epub 2012/07/19. doi: 10.1245/s10434-012-2474-4 . 22805860

[21] Lodyga M, De Falco V, Bai XH, Kapus A, Melillo RM, Santoro M, et al. XB130, a tissue-specific adaptor protein that couples the RET/PTC oncogenic kinase to PI 3-kinase pathway. Oncogene. 2009; 28(7):937–49. Epub 2008/12/09. doi: 10.1038/onc.2008.447 . 19060924

[22] Sripa B, Seubwai W, Vaeteewoottacharn K, Sawanyawisuth K, Silsirivanit A, Kaewkong W, et al. Functional and genetic characterization of three cell lines derived from a single tumor of an Opisthorchis viverrini-associated cholangiocarcinoma patient. Hum Cell. 2020; 33:695–708. Epub 2020/03/25. doi: 10.1007/s13577-020-00334-w . 32207095

[23] Kaewlert W, Sakonsinsiri C, Namwat N, Sawanyawisuth K, Ungarreevittaya P, Khuntikeo N, et al. The Importance of CYP19A1 in Estrogen Receptor-Positive Cholangiocarcinoma. Horm Cancer. 2018; 9(6):408–19. Epub 2018/10/05. doi: 10.1007/s12672-018-0349-2 . 30284180 PMC10355912

[24] Chaiprasert T, Armartmuntree N, Techasen A, Sakonsinsiri C, Pinlaor S, Ungarreevittaya P, et al. Roles of Zinc Finger Protein 423 in Proliferation and Invasion of Cholangiocarcinoma through Oxidative Stress. Biomolecules. 2019; 9(7):263. Epub 2019/07/10. doi: 10.3390/biom9070263 31284679 PMC6681239

[25] Schneider CA, Rasband WS, Eliceiri KW. NIH Image to ImageJ: 25 years of image analysis. Nat Methods. 2012; 9(7):671–5. doi: 10.1038/nmeth.2089 22930834 PMC5554542

[26] Collins TJ. ImageJ for microscopy. Biotechniques. 2007; 43(S1):S25–S30. doi: 10.2144/000112517 . 17936939

[27] Crowe AR, Yue W. Semi-quantitative Determination of Protein Expression using Immunohistochemistry Staining and Analysis: An Integrated Protocol. Bio Protoc. 2019; 9(24):e3465. Epub 2019/12/24. doi: 10.21769/BioProtoc.3465 31867411 PMC6924920

[28] Emmadi R, Wiley EL. Evaluation of resection margins in breast conservation therapy: the pathology perspective—past, present, and future. J Surg Oncol. 2012; 2012:180259. doi: 10.1155/2012/180259 23213495 PMC3507155

[29] Rosen RD, Sapra A. TNM Classification. StatPearls. Treasure Island (FL): StatPearls Publishing; 2020. 31985980

[30] Thanan R, Kaewlert W, Sakonsinsiri C, Chaiprasert T, Armartmuntree N, Muengsaen D, et al. Opposing Roles of FoxA1 and FoxA3 in Intrahepatic Cholangiocarcinoma Progression. Int J Mol Sci. 2020; 21(5):1796. Epub 2020/03/11. doi: 10.3390/ijms21051796 32151057 PMC7084256

[31] Yamanaka D, Akama T, Fukushima T, Nedachi T, Kawasaki C, Chida K, et al. Phosphatidylinositol 3-kinase-binding protein, PI3KAP/XB130, is required for cAMP-induced amplification of IGF mitogenic activity in FRTL-5 thyroid cells. Mol Endocrinol. 2012; 26(6):1043–55. Epub 2012/04/13. doi: 10.1210/me.2011-1349 22496359 PMC5416991

[32] Shi M, Zheng D, Sun L, Wang L, Lin L, Wu Y, et al. XB130 promotes proliferation and invasion of gastric cancer cells. J Transl Med. 2014; 12:1. Epub 2014/01/07. doi: 10.1186/1479-5876-12-1 24387290 PMC3882781

[33] Li J, Sun W, Wei H, Wang X, Li H, Yi Z. Expression of XB130 in human ductal breast cancer. Int J Clin Exp Pathol. 2015; 8(5):5300–8. Epub 2015/07/21. 26191231 PMC4503102

[34] Shen J, Jin C, Liu Y, Rao H, Liu J, Li J. XB130 enhances invasion and migration of human colorectal cancer cells by promoting epithelialmesenchymal transition. Mol Med Rep. 2017; 16(4):5592–8. Epub 2017/08/30. doi: 10.3892/mmr.2017.7279 . 28849225

[35] Huang M, Huang B, Li G, Zeng S. Apatinib affect VEGF-mediated cell proliferation, migration, invasion via blocking VEGFR2/RAF/MEK/ERK and PI3K/AKT pathways in cholangiocarcinoma cell. BMC Gastroenterol. 2018; 18(1):169. Epub 2018/11/08. doi: 10.1186/s12876-018-0870-3 30400838 PMC6220519

[36] Toba H, Wang Y, Bai X, Zamel R, Cho HR, Liu H, et al. XB130 promotes bronchioalveolar stem cell and Club cell proliferation in airway epithelial repair and regeneration. Oncotarget. 2015; 6(31):30803–17. Epub 2015/09/12. doi: 10.18632/oncotarget.5062 26360608 PMC4741569

[37] Moodley S, Hui Bai X, Kapus A, Yang B, Liu M. XB130/Tks5 scaffold protein interaction regulates Src-mediated cell proliferation and survival. Mol Biol Cell. 2015; 26(24):4492–502. Epub 2015/10/09. doi: 10.1091/mbc.E15-07-0483 26446840 PMC4666142

[38] Yothaisong S, Dokduang H, Techasen A, Namwat N, Yongvanit P, Bhudhisawasdi V, et al. Increased activation of PI3K/AKT signaling pathway is associated with cholangiocarcinoma metastasis and PI3K/mTOR inhibition presents a possible therapeutic strategy. Tumour Biol. 2013; 34(6):3637–48. Epub 2013/07/09. doi: 10.1007/s13277-013-0945-2 . 23832540

[39] Menakongka A, Suthiphongchai T. Involvement of PI3K and ERK1/2 pathways in hepatocyte growth factor-induced cholangiocarcinoma cell invasion. World J Gastroenterol. 2010; 16(6):713–22. Epub 2010/02/06. doi: 10.3748/wjg.v16.i6.713 20135719 PMC2817059

[40] Zhu B, Wei Y. Antitumor activity of celastrol by inhibition of proliferation, invasion, and migration in cholangiocarcinoma via PTEN/PI3K/Akt pathway. Cancer Med. 2020; 9(2):783–96. Epub 2020/01/21. doi: 10.1002/cam4.2719 31957323 PMC6970044

[41] Zhu X, Song X, Xie K, Zhang X, He W, Liu F. Osthole induces apoptosis and suppresses proliferation via the PI3K/Akt pathway in intrahepatic cholangiocarcinoma. Int J Mol Med. 2017; 40(4):1143–51. Epub 2017/09/14. doi: 10.3892/ijmm.2017.3113 28902342 PMC5593451

[42] Ke F, Wang Z, Song X, Ma Q, Hu Y, Jiang L, et al. Cryptotanshinone induces cell cycle arrest and apoptosis through the JAK2/STAT3 and PI3K/Akt/NFkappaB pathways in cholangiocarcinoma cells. Drug Des Devel Ther. 2017; 11:1753–66. Epub 2017/07/04. doi: 10.2147/DDDT.S132488 28670110 PMC5479302

[43] Chen MH, Chiang KC, Cheng CT, Huang SC, Chen YY, Chen TW, et al. Antitumor activity of the combination of an HSP90 inhibitor and a PI3K/mTOR dual inhibitor against cholangiocarcinoma. Oncotarget. 2014; 5(9):2372–89. Epub 2014/05/07. doi: 10.18632/oncotarget.1706 24796583 PMC4058012

