# Supplementary figures and images for: Adaptor protein XB130 regulates the aggressiveness of cholangiocarcinoma
Source: PLoS One. 2021 Nov 15;16(11):e0259075. doi: 10.1371/journal.pone.0259075 (PMC8592414; doi:10.1371/journal.pone.0259075)

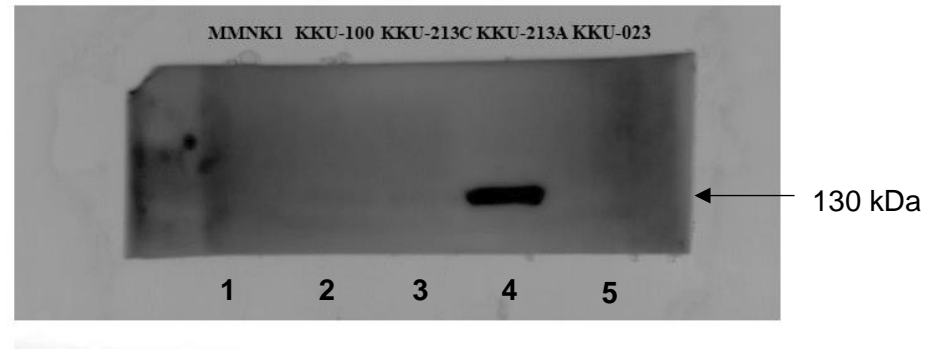

**S1 Fig. Original image of western blot result of XB130 expression in cell lines.**

Supplement: S1 Fig — (PDF) [file pone.0259075.s001.pdf]

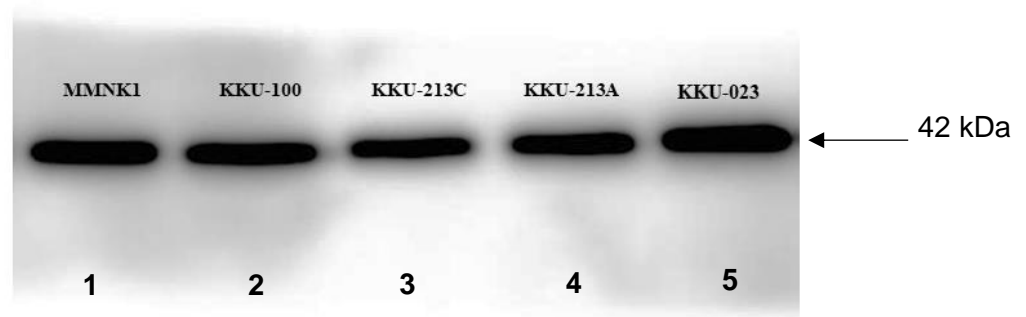

**S2 Fig. Original image of western blot result of  $\beta$ -actin expression in cell lines.**

Supplement: S2 Fig — (PDF) [file pone.0259075.s002.pdf]

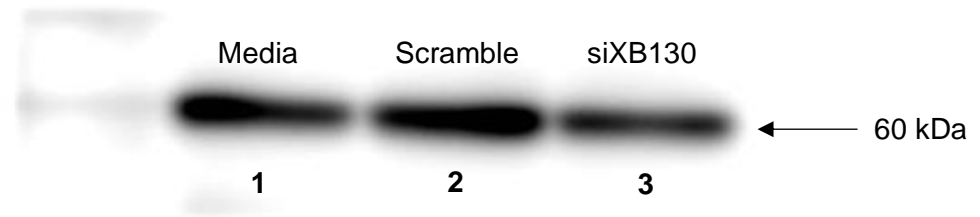

**S4 Fig. Original image of western blot result of Akt expression in media, scramble and siXB130 cells.**

Supplement: S4 Fig — (PDF) [file pone.0259075.s004.pdf]

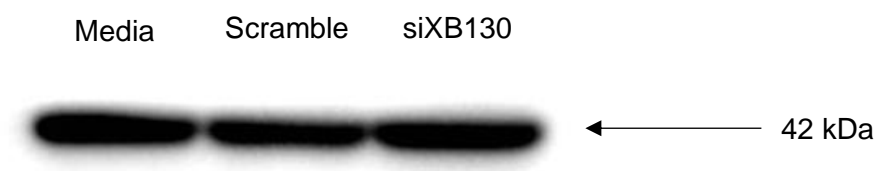

**S7 Fig. Original image of western blot result of  $\beta$ -actin in media, scramble and siXB130 cells.**

Supplement: S7 Fig — (PDF) [file pone.0259075.s007.pdf]
