# Supplementary material for: Adaptor protein XB130 regulates the aggressiveness of cholangiocarcinoma
Source: PLoS One. 2021 Nov 15;16(11):e0259075. doi: 10.1371/journal.pone.0259075 (PMC8592414; doi:10.1371/journal.pone.0259075)
Supplement: S3 Fig — (PDF) [file pone.0259075.s003.pdf]

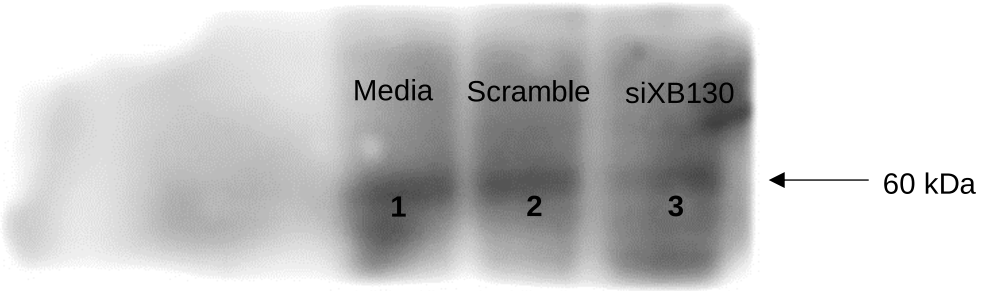

S3 Fig. Original image of western blot result of phosphorylation of Akt expression in media, scramble and siXB130 cells.
