# Supplementary material for: Adaptor protein XB130 regulates the aggressiveness of cholangiocarcinoma
Source: PLoS One. 2021 Nov 15;16(11):e0259075. doi: 10.1371/journal.pone.0259075 (PMC8592414; doi:10.1371/journal.pone.0259075)
Supplement: S8 Fig — (PDF) [file pone.0259075.s008.pdf]

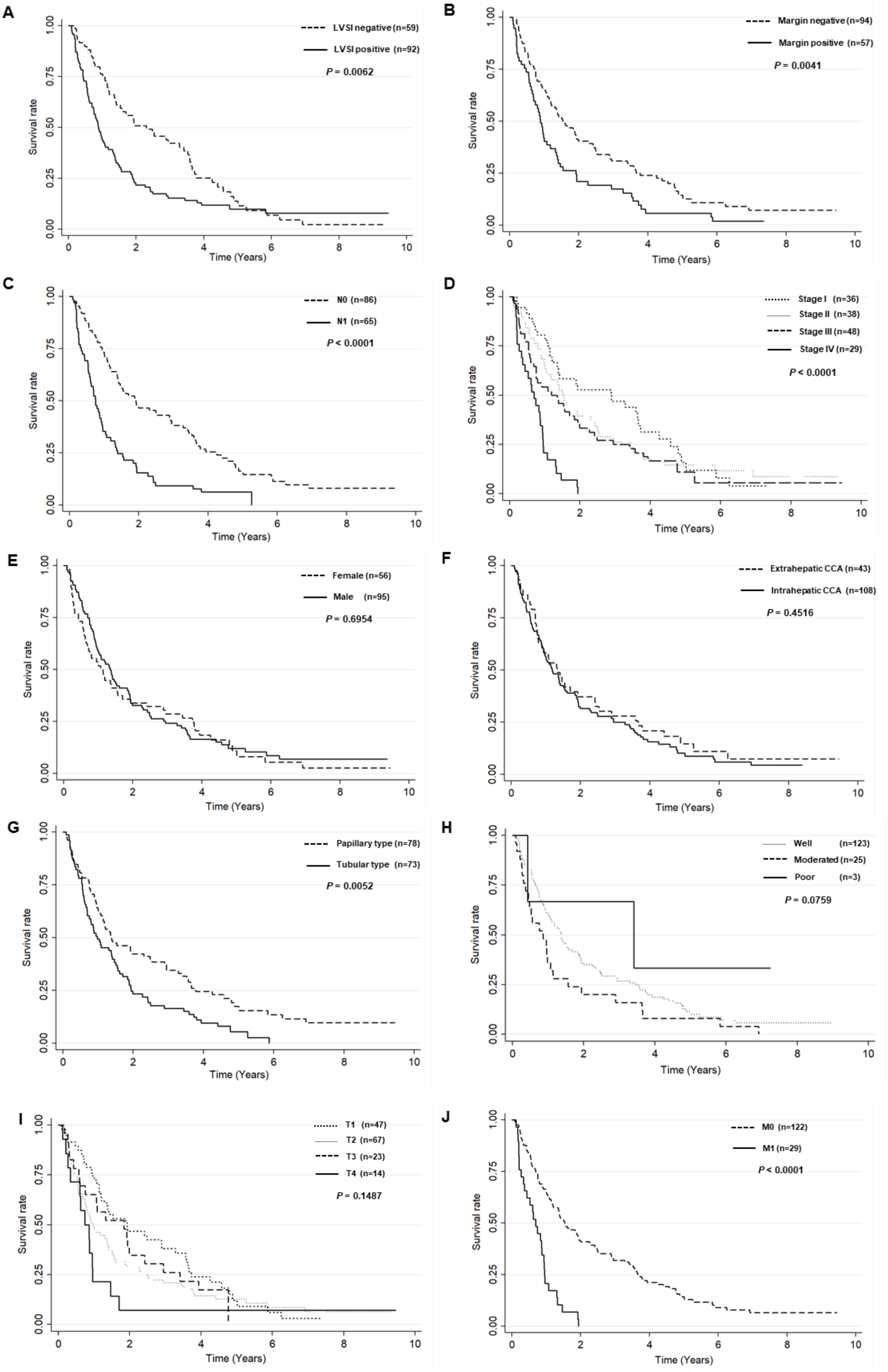
A
LVSI negative (n=59)
LVSI positive (n=92)
P = 0.0062
Survival rate
Time (Years)
B
Margin negative (n=94)
Margin positive (n=57)
P = 0.0041
Survival rate
Time (Years)
C
N0 (n=86)
N1 (n=65)
P < 0.0001
Survival rate
Time (Years)
D
Stage I (n=36)
Stage II (n=38)
Stage III (n=48)
Stage IV (n=29)
P < 0.0001
Survival rate
Time (Years)
E
Female (n=56)
Male (n=95)
P = 0.6954
Survival rate
Time (Years)
F
Extrahepatic CCA (n=43)
Intrahepatic CCA (n=108)
P = 0.4516
Survival rate
Time (Years)
G
Papillary type (n=78)
Tubular type (n=73)
P = 0.0052
Survival rate
Time (Years)
H
Well (n=123)
Moderated (n=25)
Poor (n=3)
P = 0.0759
Survival rate
Time (Years)
I
T1 (n=47)
T2 (n=67)
T3 (n=23)
T4 (n=14)
P = 0.1487
Survival rate
Time (Years)
J
M0 (n=122)
M1 (n=29)
P < 0.0001
Survival rate
Time (Years)

S8 Fig. The Kaplan-Meier analysis of clinical data. Survival curves of (S8A) LVSI, (S8B) Margin status, (S8C) N classification, (S8D) TNM staging, (S8E) Gender, (S8F) Type of CCA, (S8G) Histological type, (S8H) Histologic differentiation, (S8I) T classification, (S8J) M classification.
