# Supplementary material for: Adaptor protein XB130 regulates the aggressiveness of cholangiocarcinoma
Source: PLoS One. 2021 Nov 15;16(11):e0259075. doi: 10.1371/journal.pone.0259075 (PMC8592414; doi:10.1371/journal.pone.0259075)
Supplement: S1 Data — (PDF) [file pone.0259075.s009.pdf]

Definition of TNM staging system

For intrahepatic CCA : N0 (No regional lymph node metastasis), N1 (Regional lymph node metastasis present), M0 (N0 distant metastasis), M1 (Distant metastasis present), T1 (Solitary tumor without vascular invasion), T2 (Solitary tumor with vascular invasion), T3 (Tumor perforating the visceral peritoneum or involving the local extra hepatic structures by direct invasion), T4 (Tumor with periductal invasion), Stage I: T1+N0+M0, Stage II: T2+N0+M0, Stage III: T3+N0+M0, Stage IVA: T4+N0+M0 or Any T+N1+M0, Stage IVB: Any T+Any N+M1.

For extrahepatic CCA : N0 (No regional lymph node metastasis), N1 (Regional lymph node metastasis), M0 (N0 distant metastasis), M1 (Distant metastasis), T1 (Tumor confined to the bile duct histologically), T2 (Tumor invades beyond the wall of the bile duct), T3 (Tumor invades the gallbladder, pancreas, duodenum, or other adjacent organs without involvement of the celiac axis, or the superior mesenteric artery), T4 (Tumor involves the celiac axis, or the superior mesenteric artery), Stage I: T1-2+N0+M0, Stage II: T3+N0+M0 or T1-3+N1+M0, Stage III: T4+Any N+M0, Stage IV: Any T+Any N+M1.
