# Supplementary material for: Adaptor protein XB130 regulates the aggressiveness of cholangiocarcinoma
Source: PLoS One. 2021 Nov 15;16(11):e0259075. doi: 10.1371/journal.pone.0259075 (PMC8592414; doi:10.1371/journal.pone.0259075)
Supplement: S2 Data — (PDF) [file pone.0259075.s010.pdf]

Determination of protein expression by ImageJ

Use of Deconvolution of the IHC image using ImageJ Fiji software

- Click on the IHC image to make the image active.
- Click the “Image” option and select “Color” > “Color Deconvolution.”
- A new pop-up Color Deconvolution window will show up. For IHC images stained with 3,3’-diaminobenzidine (DAB) and hematoxylin (H), select the “H DAB” vector option. Leave “Show Matrices” and “Hide Legend” unchecked and click “Okay.”

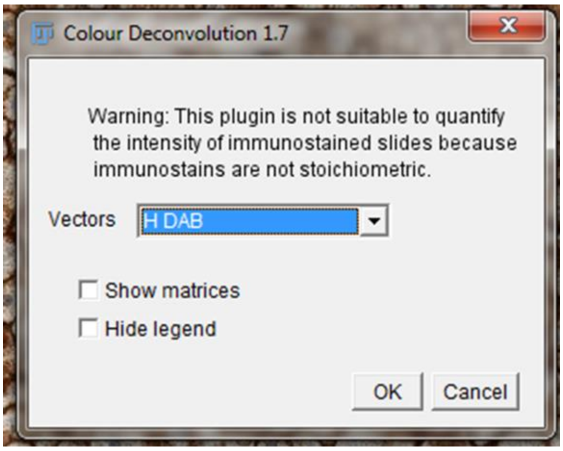

- After selecting the H DAB option, three different images will pop up on the computer screen. Colour 1 window represents only the Hematoxylin staining (blue/purple) and Colour 2 window represents only the DAB staining (brown).

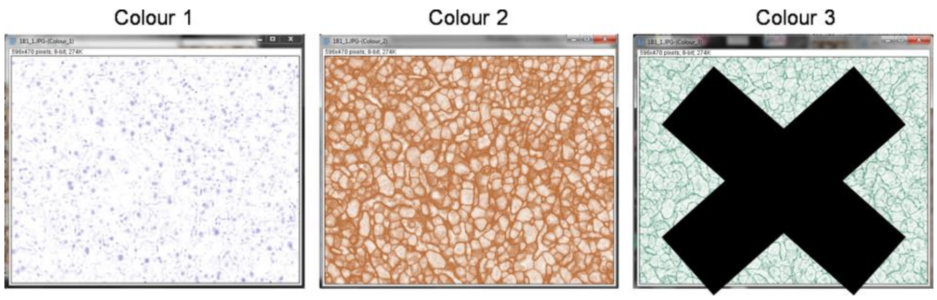

- Exit out of the third Colour 3 window, as this will not be needed for the image analysis.
- Click on the DAB Colour 2 image to activate it. DAB staining represents your primary antibody of interest.

- Go to “Image” and select “Adjust” and “Threshold.” After selecting threshold, the brown image is now converted to a black and white image.
- A new threshold window will pop up. The top bar indicates your minimum threshold value and the bottom bar indicates your maximum threshold value.
- Leave the minimum threshold value set at zero.
- Once the maximum threshold image is set, click “Apply” on the threshold window. After clicking apply, the minimum and maximum threshold values will be 255.

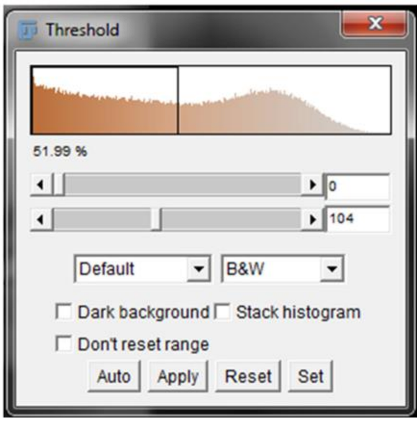

- Go to “Analyze” and select “Measure.”
