# Supplementary material for: Adaptor protein XB130 regulates the aggressiveness of cholangiocarcinoma
Source: PLoS One. 2021 Nov 15;16(11):e0259075. doi: 10.1371/journal.pone.0259075 (PMC8592414; doi:10.1371/journal.pone.0259075)
Supplement: S1 Appendix — (PDF) [file pone.0259075.s011.pdf]

APPENDIX A

Reagents for Laboratory Experiment

RIPA lysis buffer

Component	Final conc.	Amount
5 M NaCl	150 mM	0.3 ml
0.5 M Tris-HCl pH 7.4	50 mM	0.5 ml
Triton X-100	1 % (v/v)	0.1 ml
Sodium deoxycholate	1 % (w/v)	0.1 g
10% SDS	0.1 % (w/v)	0.1 ml
Distilled water		9 ml
Total		10 ml

Protease inhibitor cocktail 1 tablet was added into 10 ml mixture solution and mixed well.
