# Supplementary material for: Adaptor protein XB130 regulates the aggressiveness of cholangiocarcinoma
Source: PLoS One. 2021 Nov 15;16(11):e0259075. doi: 10.1371/journal.pone.0259075 (PMC8592414; doi:10.1371/journal.pone.0259075)
Supplement: Attachment — Submitted filename: Response to Reviewers.docx [file pone.0259075.s012.docx]

**Additional Editor Comments**

The investigation is original and quite interesting but there are important points to be considerably improved before the paper can be considered for publication:

The authors categorized a lot of variables initially expressed as cardinal numbers (which causes an important loss of information and may induce considerable bias). In particular, the immunohistochemical quantification should also be expressed in cardinal numbers and not dichotomized.

**Reply to Additional Editor Comments:**

Thank you very much for your constructive criticism and valuable suggestions for improving the quality of our manuscript. For this purpose, the expression values of XB130 were categorized into quartiles and examined by the Kaplan-Meier method followed by the log-rank test. The Kaplan-Meier survival curves showed the comparison between the survival rate of CCA patients (151 cases) and the expression levels of XB130, which were divided into quartiles (Q1-Q4) (Figure 4 of the revised manuscript). We have re-analyzed our data using cardinal numbers, including expression of XB130, TNM staging and T classification, instead of using dichotomous variables. The results showed that the Q3 of XB130 expression was significantly correlated with Stage III. Whereas, the Q4 of XB130 expression was significantly correlated with lymphovascular invasion (LVSI), TNM Stages III-IV, high T classification (T3&T4) and intrahepatic CCA in Table 1 of the revised manuscript. In addition, multivariate analysis using Cox’s proportional hazards model showed that only N classification and M classification were significantly correlated with poor survival. N and M classifications can be used as independent prognostic factors. The XB130 expression not an independent prognosis factor that affects survival outcome in CCA. Moreover, the results obtained from multivariate analysis using logistic regression demonstrated that M1 and LVSI were independent factors for lymph node metastasis. The previous analysis (The previous version of manuscript) showed that high expression of XB130 was associated with increasing risks of lymph node metastasis, which was evidenced by dichotomous analysis. However, when analyzing using cardinal numbers, the elevated level of XB130 expression was not associated with the increasing risk of lymph node metastasis (Table R2).

**Table R1** Dichotomous and cardinal analyses of XB130 expression.

| **Dichotomous analysis**  **(Low vs. high XB130 expression levels)** | **Cardinal numbers**  **(Q1, Q2, Q3, Q4)** | **Table/ Figure present in**  **the revised version of manuscript** |
| --- | --- | --- |
| CCA patients with high XB130 expression levels had shorter survival than patients with low XB130 expression levels. | XB130 protein expression was not related to prognosis. |  |
| Multivariate analysis using logistic regression demonstrated that high XB130 expression and M1 were independent factors; but, LVSI was not an independent factor for lymph node. | M1 and LVSI were independent factors; but, the elevated level of XB130 was not an independent factor for lymph node metastasis. | Table 4 in the revised manuscript**.** |
| High XB130 expression was significantly correlated with LVSI, high TNM staging, T classification, N classification and lymph node metastasis. | Elevated level of the XB130 expression was correlated with LVSI, high TNM staging, high T classification and intrahepatic CCA as demonstrated in Table 1  - Q3 XB130 expression was significantly correlated with Stage III.  -Q4 XB130 expression was significantly correlated with LVSI, StagesIII-IV, high T classification (T3&T4) and intrahepatic CCA. | Table 1 in the revised manuscript**.** |

**Table R2** Univariate and multivariate analyses (Logistic regression model) of factor correlated with lymph node metastasis (N1).

| **Clinicopathologic characteristics** | **Univariate analysis** | | | **Multivariate analysis** | | |
| --- | --- | --- | --- | --- | --- | --- |
|  | **Odds ratio** | **95% CI** | ***P* value** | **Odds ratio** | **95% CI** | ***P* value** |
| **XB130 expression**  Quartiles 1  Quartiles 2  Quartiles 3  Quartiles 4 | 1.29  2.22  2.42  1.00 | 0.5912 - 2.8245  1.1506 - 4.2918  1.1351 - 5.1665  0.5384 - 1.028 | 0.520  0.017  0.022  0.073 | 2.10 | 0.9505-4.6760 | 0.066 |
| **M category (+)** | 29.84 | 6.7488-131.9561 | <0.001 | 22.69 | 5.0132-102.7809 | <0.001 |
| **LVSI (+)** | 5.32 | 2.4970-11.3455 | <0.001 | 3.26 | 1.4002-7.6353 | 0.006 |

**Responses to Reviewers**

**Reviewer 1:**

1) Very little data regarding mechanism of action is presented. The authors show that a CCA cell line with high XB130 expression has faster proliferation, more motility and more extensive invasiveness than a normal or two CCA cell lines with low XB130 expression using *in vitro* assays. The authors also show that these properties can be suppressed in the high expresser by siRNA knockdown of XB130 as compared to a scrambled control. What is not presented is why XB130 expression promotes these activities. Several pathways downstream of XB130 are mentioned in the introduction. The authors should inhibit individual downstream pathways (e.g., PI3K/Akt) to test whether such inhibition recapitulates the effect of siRNA knockdown of XB130. The authors should also over-express XB130 in MMNK1 cells and XB130-low CCA cells to test whether this expression is sufficient to confer the proliferative, motility and invasiveness characteristics of XB130-high CCA cells. There is published data indicating that XB130 can promote tumorigenesis via activation of the PI3K/Akt pathway, but it would improve the manuscript to show that in this system. As it is now, too much of the manuscript's mechanistic conclusions rest on a single CCA cell line. More controls and more mechanistic studies are needed for this aspect.

**Reply to the Comment #1:**

Thank you very much for your constructive criticism and valuable suggestions for improving the quality of our manuscript. To improve our manuscript, the molecular mechanism underlying the function of XB130 was determined in an XB130-knockdown CCA cell line. The expression levels of total Akt (Akt), phosphorylated Akt (pAkt) at Ser473 and EMT markers (E-cadherin and vimentin) were additionally investigated using Western blotting in the XB130 knockdown-CCA cell lines. The results showed that the expression levels of Akt and pAkt were decreased in the XB130- knockdown CCA cell lines, suggesting that XB130 expression is involved in the PI3K/Akt pathway. Moreover, decreased vimentin and unchanged E-cadherin expression levels were detected in the XB130-knockdown CCA cell lines compared to the control cells, suggesting that XB130 expression induced EMT processes via the induction of vimentin. XB130 binds to the p85α subunit of PI3K and activates protein kinase B (Akt). Akt is one of major downstream targets of PI3K, and its phosphorylation at Ser473 is a sensitive indicator of Akt activity [1]. The PI3K/Akt signaling pathway was constitutively activated and was involved in cancer proliferation, migration and invasion as well as induction of EMT [1, 2]. Our results confirmed that the overexpression of XB130 in CCA accelerated the cancer progression via PI3K-Akt signaling pathway. These results were added into the revised manuscript (Figure 2A, Line 269). However, the XB130 overexpression could not be performed because of time limitation. We expect that our evidence showing that XB130 is involved in PI3K-Akt signaling pathway is sufficient.

**Figure R1** The expression levels of E-cadherin, vimentin, Akt and pAkt in siXB130 and scramble groups, determined by Western blotting. $\beta$-actin was used as a loading control.

2) For use as a biomarker, the authors need to report specificity and sensitivity. Discussion should also be provided that compares the sensitivity and specificity of XB130 against existing CCA biomarkers of which there are now a great number.

**Reply to the Comment #2:**

We apologize for our carelessness and thank the reviewer for pointing this out. We have reported the specificity and sensitivity of parameters and survival rates in the table below. The sensitivity and specificity of XB130 were similar to other parameters that showed high sensitivity but low specificity.

| **Parameter** | **%** | **95% conf. Inter.** |
| --- | --- | --- |
| **XB130** |  |  |
| Sensitivity | 88.16 | 83.00-93.31 |
| Specificity | 8.00 | 3.67-12.33 |
| **LVSI** |  |  |
| Sensitivity | 90.22 | 85.48 - 94.96 |
| Specificity | 10.17 | 5.35 – 14.99 |
| **Histology type** |  |  |
| Sensitivity | 84.62 | 78.86 - 90.37 |
| Specificity | 4.11 | 0.94 - 7.28 |
| **Lymph node metastasis** |  |  |
| Sensitivity | 92.31 | 88.06 - 96.56 |
| Specificity | 12.33 | 7.08 - 17.57 |
| **Surgical margins** |  |  |
| Sensitivity | 96.49 | 93.56 - 99.43 |
| Specificity | 13.83 | 8.32 - 19.34 |
| **TNM staging** |  |  |
| Sensitivity | 89.47 | 82.48 - 96.47 |
| Specificity | 13.89 | 6.01 - 21.77 |
| **N classification** |  |  |
| Sensitivity | 95.38 | 92.04 - 98.73 |
| Specificity | 13.95 | 8.43 - 19.48 |
| **M classification** |  |  |
| Sensitivity | 100.00 | 100.00 - 100.00 |
| Specificity | 12.30 | 7.06 - 17.53 |

3) Table 1 should include definitions of staging features:

N0 (No regional lymph node metastasis)
N1 (Regional lymph node metastasis)
M0 (No distant metastasis)
M1 (Distant metastasis)
T1 (Solitary tumor; no vascular invasion)
T2a (Solitary tumor with vascular invasion)
T2b (Multiple tumors)
T3 (Tumor that has perforated the visceral peritoneum or otherwise demonstrating invasivity)
T4 (Tumor with periductal invasion).
Stage I: T1+N0+M0
Stage II: T2+N0+M0
Stage III: T3+N0+M0
Stage IVA: T4+N0+M0
Stage IVB: T(1-4)+N1 or T(1-4)+M1

**Reply to the Comment #3:**

We thank the Reviewer for this comment and suggestion. To the revised manuscript, we have added the definitions of staging features in Supplementary file#1.

**Reviewer 2:** The authors analyze the expression of the adaptor protein XB130 in 151 cases with CCA. They note poor survival rates in cases with high XB 130 expression, which they confirm by studies of 4 cell lines. This is a good article and should be published provided that

1. The discrepancies in table 1 (M0, M1<> TNM); (N0, N1<> Lymph node metastases) etc. should be explained 7 corrected.

**Reply to the comment#1:**

We thank the reviewer for pointing out this concern. To improve our manuscript, we have explained definition of TNM staging system as described as follows:

**For intrahepatic CCA** : N0 (No regional lymph node metastasis), N1 (Regional lymph node metastasis present), M0 (N0 distant metastasis), M1 (Distant metastasis present), T1 (Solitary tumor without vascular invasion), T2 (Solitary tumor with vascular invasion), T3 (Tumor perforating the visceral peritoneum or involving the local extra hepatic structures by direct invasion), T4 (Tumor with periductal invasion), Stage I: T1+N0+M0, Stage II: T2+N0+M0, Stage III: T3+N0+M0, Stage IVA: T4+N0+M0 or Any T+N1+M0, Stage IVB: Any T+Any N+M1.

**For extrahepatic CCA** : N0 (No regional lymph node metastasis), N1 (Regional lymph node metastasis ), M0 (N0 distant metastasis), M1 (Distant metastasis ), T1 (Tumor confined to the bile duct histologically), T2 (Tumor invades beyond the wall of the bile duct), T3 (Tumor invades the gallbladder, pancreas, duodenum, or other adjacent organs without involvement of the celiac axis, or the superior mesenteric artery), T4 (Tumor involves the celiac axis, or the superior mesenteric artery), Stage I: T1-2+N0+M0, Stage II: T3+N0+M0 or T1-3+N1+M0, Stage III: T4+Any N+M0, Stage IV: Any T+Any N+M1.

2. What about pTNM? Tumor size, organ of metastasis (liver, others?)

**Reply to the comment#2:**

We thank the reviewer for this constructive comments and suggestion. To the revised manuscript, we have added the following

**For pTNM of intrahepatic CCA** : N0 (No regional lymph node metastasis), N1 (Regional lymph node metastasis present), M0 (N0 distant metastasis), M1 (Distant metastasis present), T1 (Solitary tumor without vascular invasion), T2 (Solitary tumor with vascular invasion), T3 (Tumor perforating the visceral peritoneum or involving the local extra hepatic structures by direct invasion), T4 (Tumor with periductal invasion), Stage I: T1+N0+M0, Stage II: T2+N0+M0, Stage III: T3+N0+M0, Stage IVA: T4+N0+M0 or Any T+N1+M0, Stage IVB: Any T+Any N+M1.

**For pTNM of extrahepatic CCA** : N0 (No regional lymph node metastasis), N1 (Regional lymph node metastasis ), M0 (N0 distant metastasis), M1 (Distant metastasis ), T1 (Tumor confined to the bile duct histologically), T2 (Tumor invades beyond the wall of the bile duct), T3 (Tumor invades the gallbladder, pancreas, duodenum, or other adjacent organs without involvement of the celiac axis, or the superior mesenteric artery), T4 (Tumor involves the celiac axis, or the superior mesenteric artery), Stage I: T1-2+N0+M0, Stage II: T3+N0+M0 or T1-3+N1+M0, Stage III: T4+Any N+M0, Stage IV: Any T+Any N+M1.

Regarding the organ of metastasis, a total number of metastatic cases were 87 out of 151 cases. Of this population, liver (57.9%) was the most common site of ICC metastases, followed by lung, bone, spleen, brain, intestine and gastric.

3. The collection time (2007 - 2017) is quite long; what about changes of the TNM classification during that time?

**Reply to the comment#3:**

We appreciate the reviewer’s insightful and helpful comment and apologize for any confusion. In this study, we used the 7^th^ edition (2010-2017) of the AJCC Cancer Staging for classification of TNM staging. The patients diagnosed in 2007-2009 underwent re-staging according to the criteria of 7^th^ edition.

4. The aspect of tumor cell heterogeity should be briefly addressed.

**Reply to the Comment #4:**

We appreciate the reviewer’s concern about tumor cell heterogeneity. We performed an additional experiment on the tumor cell heterogeneity. The XB130 immunohistochemistry staining on the whole sections of 3 cases were quite homogenous as shown in Figure R2. Regarding the homogenous expression of XB130 expression the CCA, the TMA cores might reflect scores obtained from whole sections.

**CASE 1 (Quartile 2)**

**CASE 2 (Quartile 3)**

**CASE 3 (Quartile 4)**

**Figure R2** XB130 immunohistochemistry staining on the whole sections of 3 cases.

5. The IHC color classification threshold / method should be explained more in detail (which color space, which color adjustment / image quality analysis has been performed.

**Reply to the comment#5:**

We apologize for not having discussed it clearly. We added more detail on the data analysis as shown in the Supplementary file#2. We hope that changes made in the updated manuscript will help to clarify our statement.

**Reviewer 3:** The study by Poosekeaw et al, deals with the role of the adaptor protein XB130 in cholangiocarcinoma. The authors present data using 5 different cell lines, and one of them expressing XB130. For further experiments, they have used XB130-expressing cell line and via siRNA they knocked down XB130. They could show that XB130 plays a role in proliferation, migration and invasion, at least in this cell line. Additional, they have analysed XB130 expression in 151 CCA tissue samples and could find a correlation of XB130 expression, M classification and LVSI. Although of interest, following improvements have to be done before it can be considered for publication in Plos One.

Critical_points:
1) Figure 1: First, the authors should mention in the fig-legend that tubulin serves as a loading control. Second, please indicate kDa of target protein in brackets. Furthermore, it would also be interesting to know why the other cell do not express XB130 at all. Have the authors checked mRNA expression? Is there a post-transcriptional modification? Are there any mutations or epigenetically silencing in/of XB130 known?

**Reply to the Comment #1:**

We thank the reviewer for valuable comments. We have added the loading control (β-actin) in Figure 1 and indicated kDa of the target protein in brackets in the revised manuscript (Fig 1, Line 245). To address the question why other cells did not express XB130 at all, we have attempted to search in in the literature for further information on the mutation and epigenetic changes of XB130 gene in CCA; we however could not find any. It would be would be very interesting to study on mutation and epigenetics of this gene which could be potential directions for future research.

We deeply apologize for the lack of clarifying in term of XB130 mRNA expression in CCA cell lines and MMNK1 cell line in this study. However, we sought to investigate the XB130 mRNA expression levels in CCA tissues (CHOL, n = 36) and normal bile duct tissues (n = 9) using Pan-cancer analysis [3]. The XB130 mRNA levels were significantly higher in CCA tissues (red box) than in normal tissues (black box). These data suggested that XB130 was over-expressed in CCA tissues. According to these data, we decided to determine only protein expression in both CCA cell lines and tissues.

**Figure R3**	XB130 mRNA expression levels in cancer (red box) and normal (black box) tissues from GEPIA database. CHOL abbreviates for cholangiocarcinoma (CCA). CCA tissues (n = 36) and normal bile duct tissues (n = 9).

2) Figure 2: General for Fig2A-F. It would be a nice control to see all parameters in untreated KKU-213A (meaning without any transfection). Next point: the authors show a complete down regulation after 48h but, if I understood the data right, the results of proliferation, migration and invasion were analyzed after additional 24h? In order to check the efficiency of the XB130 knock down a time course would be helpful – Fig2A: Western Blot after 24, 48 and 72h post-transfection. An additional control experiment would be to use a XB130 non-expressing cell line (e.g. KKU213C).

**Reply to the Comment #2:**

We thank the reviewer for this helpful comment and suggestion. To validate our results, we optimized the transfection time before performing other experiments. We checked the expression levels of XB130 in the CCA cell lines (media, scramble and siXB130) at 24, 48 and 72 h after transfection as shown in Figure R4. The siRNA clearly reduced XB130 expression from 24-72 h after transfection. Scrambled siRNAs were used as vehicle controls.

**Figure R4** The expressions of XB130 (130 kDa) in media, siXB130 and scramble after 24, 48 and 72 h post-transfection.

3) Figure 3: Please give the scale bar! Furthermore, it would be helpful to mark the CCA cells (e.g. with a *), normal bile duct (e.g. with an arrow) and hepatocytes (e.g. with an arrowhead) for non-histologists.

**Reply to the Comment #3:**

We are very grateful for insightful comments and suggestions. Each image contain only normal bile duct cells (Figure 3A), hepatocytes (Figure 3B), and cancer cells (Figures 3C-F). XB130 protein was highly detected in cytoplasm of cancer cells, whereas it was slightly detected in normal bile duct cells and hepatocytes. We added the IHC results for normal bile duct cells (Fig 3A) and hepatocytes (Figure 3B) as well as scale bar in Figure 3C of the revised manuscript.

4) Fig 4, legend: please indicate the number of patients (n)

**Reply to the Comment #4:**

Thank you very much for this helpful comment. We have added the number of patients in the revised manuscript and highlighted in legend (Fig 4 in the revised manuscript).

5) Have the authors checked for downstream targets, like PI3K or Akt? These experiments could be easily done by Western Blotting and would underline the specific role of XB130 in tested cell lines and in FFPE material (in this case testing by IHC).

**Reply to the Comment #5:**

We thank the reviewer for pointing out this major concern. We have investigated downstream targets of XB130, such as Akt, phosphorylated form of Akt (pAkt) at Ser473 E-cadherin and vimentin. The results showed that the expression levels of both Akt and pAkt were decreased in the XB130-knockdown CCA cell lines, suggesting that XB130 expression involved in PI3K/Akt pathway (Figure R5). Moreover, decreased vimentin and unchanged E-cadherin expression levels were detected in the XB130-knockdown CCA cell lines compared to the control cells, suggesting that XB130 expression induces EMT processes via the induction of vimentin. XB130 binds to the p85α subunit of PI3K and activates protein kinase B (Akt) [1]. Akt is a downstream target of PI3K and its phosphorylation at Ser473 is a sensitive indicator of Akt activity [4]. The PI3K/Akt signaling pathway was constitutively activated and was involved in cancer proliferation, migration and invasion as well as induction of EMT [1, 2]. Our results confirmed that the overexpression of XB130 in CCA accelerates the cancer progression via PI3K-Akt signaling pathway. These results were added into the revised manuscript as Figure 2A. Scramble siRNAs (scramble) were used as vehicle controls. In this present work, we performed migration and invasion assays in the scramble control cells and siXB130 cells; unfortunately, the experiments on the migration and invasion in media have not been done because of research funding limitations.

**Figure R5** The expression levels of XB130 (130 kDa), E-cadherin (~135 kDa), vimentin (~54 kDa), Akt (~60 kDa) and pAkt (~60 kDa) in siXB130 and scramble groups, determined by Western blotting. $\beta$-actin was used as a loading control.

6) A further suggestion/question: are data available for Ki67 level in the FFPE samples?

**Reply to the Comment #6:**

We thank the reviewer for raising this critical issue. To respond to the reviewer’s concern, we have planned to detect Ki67 expression levels in tissues; however, the experiment could not be performed because we have had limited tissue sections in TMA blocks as they were also used in previously completed projects. We then sought to investigate the expression levels of XB130 and Ki67 in CCA and normal bile duct tissues using Pan-cancer analysis [3]. It was found that the expression levels of Ki-67 were not significantly correlated with XB130 (p = 0.17, R = 0.21). Low number of cases for both CCA (n = 36) and normal bile duct (n = 9) tissues may not give the difference that have sufficient power to detect a significant difference between the two groups as shown in Figure R6. However, according to the Pan-cancer analysis, the graph showed an increasing trend of XB130 expression levels, which was associated with high levels of Ki67. These data supported that the XB130 expression levels can affect cell proliferation.

**Figure R6** The correlation of XB130 (AFAP1L2) and Ki67 (MKI67) mRNA expression levels in CCA and normal bile duct tissues using Pan-cancer analysis.

7) Have the authors checked, both cell lines (WB) and FFPE (IHC), EMT-related targets (N-, E-cadherin, vimentin, or EMT-TFs, e.g. TWIST, SNAI, ZEB)? I think these experiments would improve the paper and underline the conclusions the authors have done!

**Reply to the comment#7:**

We thank the reviewer for this insightful comment and suggestion. We measured E-cadherin and vimentin expression levels for EMT markers. The results are previously shown in Figure R5. Decreased vimentin and unchanged E-cadherin expression levels were detected in the XB130-knockdown CCA cell lines compared to the control cells. These finding suggest that XB130 expression induces EMT processes via the induction of vimentin.

**References**

1.	Shiozaki A, Shen-Tu G, Bai X, Iitaka D, De Falco V, Santoro M, et al. XB130 mediates cancer cell proliferation and survival through multiple signaling events downstream of Akt. PLoS One. 2012; 7(8):e43646. Epub 2012/08/29. <http://doi.rog/10.1371/journal.pone.0043646>. PMID: 22928011; PubMed Central PMCID: PMCPMC3426539.

2.	Yamanaka D, Akama T, Fukushima T, Nedachi T, Kawasaki C, Chida K, et al. Phosphatidylinositol 3-kinase-binding protein, PI3KAP/XB130, is required for cAMP-induced amplification of IGF mitogenic activity in FRTL-5 thyroid cells. Mol Endocrinol. 2012; 26(6):1043-55. Epub 2012/04/13. <http://doi.rog/10.1210/me.2011-1349>. PMID: 22496359; PubMed Central PMCID: PMCPMC5416991.

3.	Tang Z, Li C, Kang B, Gao G, Li C, Zhang Z. GEPIA: a web server for cancer and normal gene expression profiling and interactive analyses. Nucleic acids research. 2017; 45(W1):W98-W102.

4.	Alessi DR, Andjelkovic M, Caudwell B, Cron P, Morrice N, Cohen P, et al. Mechanism of activation of protein kinase B by insulin and IGF-1. The EMBO journal. 1996; 15(23):6541-51.
