# Supplementary material for: Adaptor protein XB130 regulates the aggressiveness of cholangiocarcinoma
Source: PLoS One. 2021 Nov 15;16(11):e0259075. doi: 10.1371/journal.pone.0259075 (PMC8592414; doi:10.1371/journal.pone.0259075)
Supplement: Attachment — Submitted filename: Response to Editor.docx [file pone.0259075.s013.docx]

**Response to Editor**

“***Adaptor protein XB130 regulates the aggressiveness of cholangiocarcinoma***” (Manuscript ID: PONE-D-20-37191) by Pirawan Poosekeaw *et al*.

**Academic Editor´s comments:**

1. I am not satisfied with the changes. There are still variables, measured originally in cardinal number, but tater categorized, such as age. Why is there a cut point at 50 years, instead of 45, 47, 55 etc.? Before applying quartiles, the authors should calculate with the cardinal numbers of raw data This applies to the correlations, as well as to Cox and logistic regressions!

**Reply to Additional Editor Comments#1:**

We are grateful to the Editor for your helpful comments and suggestions, which have been very useful in improving our manuscript. We also would like to deeply apologize for the confusion. First, we agreed with you that there are still variables, such as age, which were originally measured in cardinal number, but later categorized. Therefore, similar to the analysis of XB130 expression, another variable, *i.e*., age has been re-analyzed by utilizing cardinal numbers in our raw data, while the clinicopathologic parameters of CCA patients have been analyzed using linear regression (Table 1) and Cox regression (Table 2). Following the re-analyses, the Abstract, Materials and Methods, Results, and Discussion Sections in the revised manuscript were edited accordingly and marked in red as described as follows:

i) On page 2, Line 35-37, the sentence “Moreover, elevated expression levels of XB130 were significantly correlated with intrahepatic type of CAA and aggressive parameters including lymphovascular space invasion (LVSI), higher T-classification, and higher tumor staging in TNM classification.” has been changed to “Moreover, elevated XB130 expression levels were significantly correlated with lymphovascular space invasion (LVSI), intrahepatic type of CCA, stage IV of TNM staging, T4 classification and lymph node metastasis.”;
ii) On page 6, Line 145-146, the Materials and Methods Section has been revised by adding the sentence “Original images of Western blot results are shown in Fig S1-S7;
iii) On page 9, Line 199-200, the Materials and Methods Section, in particular Evaluation of immunohistochemical staining, has been revised. The sentence “In the present study, we defined cut-points of the grading of XB130 expression by using the criteria of the quartile, the values for the quartiles of XB130 expression were assessed, and examined by the Kaplan–Meier method followed by the log-rank test to see a different survival.” was replaced by “XB130 expression levels (IHC scores) of 151 CCA tissues examined ranged from 28.00 to 220.45”;
iv) On page 10, Line 222-223, the sentence “The correlation between XB130 expression levels with clinicopathological features were performed by Chi-square test (𝑥^2^ *t*𝑒𝑠𝑡)” has been rewritten to “The correlation between XB130 expression levels with clinicopathological features were performed by linear regression.”;
v) On page 12, Line 283-284, the sentence “Kaplan–Meier survival curves according to the quartiles (The average pixel intensity of IHC image) of CCA tissues, 25.2% (38/151) of Q1, 25.2% (38/151) of Q2, 24.4% (37/151) of Q3 and 25.2% (38/151) of Q4. The representative results of normal bile duct, hepatocyte and CCA tissues with XB130 expression levels were showed in Fig 3.” has been replaced by the sentence “The representative results of normal bile duct, hepatocyte and CCA tissues with XB130 expression levels were shown in Fig 3.”;
vi) On page 13, Line 293-299, the Results Section: Correlation between the XB130 expression levels and clinicopathological data of CCA patients has been revised by re-analyzed the correlation between the XB130 expression levels and clinicopathological data of CCA patients using linear regression;
vii) On page 13-15, we have modified Table 1 and Table 2 in the revised manuscript and highlighted in red;
viii) On page 14, Line 311, 316, 318-320, 324-331 and 333, the Results Section: Univariate and multivariate analyses of clinicopathological variables influencing survival and lymph node metastasis in CCA has been revised by adding following words; (1) histological type, (2) tubular histological type, (3) positive margin status, (4) N1 classification, (5) M1 classification, (6) age, (7) intrahepatic type of CCA and the sentence “T classification (T2, HR = 1.3788, *P* = 0.111), T classification (T3, HR = 1.1805, *P* = 0.543), TNM staging (stage II, HR = 1.2338, *P* = 0.400), TNM staging (stage III, HR = 1.4768, *P* = 0.101), quartile 2 XB130 expression (HR = 0.7163, *P* = 0.164), quartile 3 XB130 expression (HR = 1.2646, *P* = 0.334), quartile 4 XB130 expression (HR = 0.9830, *P* = 0.944) (Table 2).” has been rewritten “XB130 expression (HR = 1.000, P = 0.356) (Table 2). The Kaplan-Meier survival curves showed the comparison of the survival rate in 151 cases of CCA patients between gender, histological type, type of CCA, M classification, LVSI, margin status, histologic differentiation, TNM staging, T classification and N classification. Tubular histological type (P = 0.0052), M1 classification (P < 0.0001), LVSI (P = 0.0062), positive margin status (P = 0.0041), stage IV of TNM staging (P < 0.001) and N1 classification (P < 0.001) were significant related to prognosis as shown in Fig S8.”;
ix) On page 19-20, Line 391-399, the sentence “In CCA tissues, elevated level of XB130 expression by IHC was associated with intrahepatic type of CCA, high T-classification (size and local invasion), high TNM staging, and lymphovascular invasion.” has been rewritten to “In CCA tissues, elevated level of XB130 expression by IHC was associated with LVSI, intrahepatic type of CCA, stage IV of TNM staging, T4 classification, and N1 classification (lymph node metastasis). The T4 classification, LVSI, lymph node metastasis, and high TNM staging all indicate tumor extension and invasion, which supported the *in vitro* investigation of cell proliferation and invasion in KKU-213A cells. The Kaplan-Meier survival curves showing LVSI, margin status, TNM staging, and N classification were significantly related to prognosis. Although, XB130 level was not directly associated with survival outcome in CCA patients, but it was associated with other factors correlated with survival, including LVSI, high TNM staging and lymph node metastasis.”;

Furthermore, in raw data, a logistic regression analysis of XB130 expression levels was performed using cardinal numbers. However, there were no statistically significant differences between lymph node metastasis (Table R1). Therefore, we decided to not include Table R1 in the revised manuscript.

**Table R1.** Univariate and multivariate logistic regression analyses of factors that are related to lymph node metastasis (N1).

| **Clinicopathologic characteristic** | **Univariate** | | | **Multivariate** | | |
| --- | --- | --- | --- | --- | --- | --- |
|  | **Odds ratio** | **95% CI** | ***P* value** | **Odds ratio** | **95% CI** | ***P* value** |
| **M category (+)** | 29.84 | 6.7488 - 131.9561 | <0.001 | 22.17 | 4.9082 - 100.1789 | <0.001 |
| **LVSI (+)** | 5.32 | 2.4970 - 11.3455 | <0.001 | 3.69 | 1.6187 -8.4202 | 0.002 |
| **XB130** | 1.00 | 1.0018 – 1.0160 | 0.013 | 1.0074 | 0.9990 – 1.0158 | 0.083 |

In response to your question regarding an age cut-off, 50 years old was used as the age cut-off in the previous version of this manuscript following the age cut-off in the previous report by Farhat MH *et al.* for survival analysis of CCA patients. (Farhat, Shamseddine et al. 2008). However, we excluded the data in the revised manuscript. In the revised manuscript, we reanalyzed the data using cardinal numbers of ages instead of an age cut-off of 50 years old.

**Reference:**

Farhat, M. H., et al. (2008). "Prognostic factors in patients with advanced cholangiocarcinoma: role of surgery, chemotherapy and body mass index." World Journal of Gastroenterology: WJG 14(20): 3224.

2. The graphic in the reply to the editor should be integrated into the manuscript.  Regarding this graphic please try non-linear curve-fittings, perhaps a logarithmic or, perhaps still better, a hyperbolic function and give the R squares as estimates of goodness-of-fit!. Please discuss this result!

**Reply to Additional Editor Comments#2:**

We thank the Editor for pointing this out. We have re-analyzed our raw data by calculating the correlation of XB130 expression using cardinal numbers and 5-year survival probability of CCA patients (Fig. R1). Unfortunately, this analysis did not provide any clear evidence of an association between XB130 expression and 5-year survival probability.

**Figure R1** A scatter plot between XB130 expression (intensity) and 5-year survival probability of CCA patients.

We further attempted to operate the logarithmic (Fig. R2), inverse (Fig. R3), cubic (Fig. R4) and square root (Fig. R5) functions of the XB130 expression. However, a significant correlation between XB130 expression and 5-year survival probability of CCA patients was not found.

**Figure R2** A scatter plot representing the relationship between the logarithm of XB130 expression (log 10) and 5-year survival probability of CCA patients.

**Figure R3** A scatter plot representing the relationship between the inverse XB130 expression and 5-year survival probability of CCA patients.

**Figure R4** A scatter plot between cubic function of XB130 expression and 5-year survival probability of CCA patients.

**Figure R5** A scatter plot between square root of XB130 expression and 5-year survival probability of CCA patients.

We further sought to perform subgroup analyses using cardinal numbers of raw data of XB130 expression in the patients with different histology types (tubular and papillary types), N classification (N0 and N1), and M classification (M0 and M1) subgroups as shown in Fig. R6A-F. In all subgroups, there were still no statistically significant association between XB130 expression and 5-year survival probability.

**Figure R6** A scatter plot representing between: A) tubular type, B) papillary type, C) N0, D) N1, E) M0, F) M1 and 5-year survival probability of CCA patients.

XB130 level was not directly associated with survival outcome in CCA patients (Fig. R1-R6). As a result, after many attempts to re-analyze our results, we are afraid that Fig. R1-R6 will not be included in the revised manuscript. However, our results in cell line and CCA tissues strongly supported that the overexpression of XB130 in the CCA cell line was related to cell proliferation, migration and invasion, leading to significantly associate with the progression of CCA. Moreover, elevated level of XB130 expression by IHC was associated with LVSI, intrahepatic type of CCA, stage IV of TNM staging, T4 classification, and N1 classification (lymph node metastasis), which can support the *in vitro* investigation of cell proliferation and invasion in KKU-213A cells.
