# Supplementary material for: Adaptor protein XB130 regulates the aggressiveness of cholangiocarcinoma
Source: PLoS One. 2021 Nov 15;16(11):e0259075. doi: 10.1371/journal.pone.0259075 (PMC8592414; doi:10.1371/journal.pone.0259075)
Supplement: Attachment — Submitted filename: Response to Editor.docx [file pone.0259075.s014.docx]

**Response to Editor**

“***Adaptor protein XB130 regulates the aggressiveness of cholangiocarcinoma***” (Manuscript ID: PONE-D-20-37191) by Pirawan Poosekeaw *et al*.

**Academic Editor´s comment:**

1.The paper has been improved. Problems are still found in table 1. Are these data really all correlations? Which kind of correlation? Spearman? Kendall? Pearson? The correlation coefficients are all missing! Please add the coefficients together with their confidence intervals in all cases with p<0.05

**Response to Editor’s comment:**

We are truly grateful for your consideration of this manuscript. We also very much appreciate your comments and suggestions that helped improving our manuscript. In the previous version of our revised manuscript, we analyzed the data using linear regression as shown in Table R1-1. However, in the previous version, we sincerely apologize for not having included correlation coefficients, 95% confidence intervals, and *β* values in it. This was clearly a mistake on our part. In this new version of our revised manuscript, we added correlation coefficients, 95% CI, and *β* values in Table 1 in the recent version of our revised manuscript, which is also given below. Newly added values are marked in red.

**Table R1-1.** Correlation between XB130 expression and clinicopathologic features of CCA patients.

| **Variable** | **XB130 expression** |
| --- | --- |
|  | ***P-*value** |
| Age | 0.976 |
| Gender | 0.958 |
| LVSI | 0.005 |
| Histological type | 0.749 |
| Margin status | 0.110 |
| Type of CCA | 0.033 |
| Histologic Differentiation | 0.072 |
| TNM staging | < 0.001 |
| T classification | < 0.001 |
| N classification | 0.011 |
| M classification | 0.365 |

*Definition of TNM staging system described in S1

**Table 1.** Relationship between XB130 expression and clinicopathologic features of CCA patients.

| **Variable** | **XB130 expression** | | | |
| --- | --- | --- | --- | --- |
|  | ***Coef.*** | ***95% CI*** | ***β*** | ***P-*value** |
| **Gender**  Female/male | -0.12 | -8.9111 - 8.6517 | 0.0042 | 0.976 |
| **Age**  Years | 0.41 | -15.4027 - 16.2285 | -0.0023 | 0.958 |
| **LVSI**  Yes/No | 22.08 | 6.7806 - 37.3836 | 0.2274 | 0.005 |
| **Histological type**  Tubular/Papillary type | 2.48 | -12.8509 - 17.8225 | 0.0262 | 0.749 |
| **Margin status**  Yes/No | -12.75 | -28.4395 - 2.9211 | -0.1305 | 0.110 |
| **Type of CCA**  Intrahepatic/Extrahepatic | 17.03 | 0.2762 - 33.8017 | 0.1623 | 0.046 |
| **Histologic Differentiation**  Well  Moderated  Poor | 15.78  50.16 | 1  -4.5935 - 36.1560  -4.1080 - 104.4313 | 0.1238  0.1477 | 0.128  0.070 |
| **TNM staging**  I  II  III  IV | 10.58  41.17  28.41 | 1  -10.0906 - 31.2698  21.5696 - 60.7782  6.2269 - 50.6001 | 0.0970  0.4048  0.2363 | 0.313  < 0.001  0.012 |
| **T classification**  T1  T2  T3  T4 | 3.93  56.87  54.32 | 1  -11.8204 - 19.6827  35.8058 - 77.9391  29.1228 - 79.5356 | 0.0412  0.4314  0.3327 | 0.623  < 0.001  < 0.001 |
| **N classification**  N1/N0 | 19.71 | 4.5583 - 34.8619 | 0.2060 | 0.011 |
| **M classification**  M1/M0 | 8.91 | -10.494 - 28.3251 | 0.074 | 0.365 |

*Definition of TNM staging system is described in S1

**Coef.,* coefficients; *95% CI*, 95% confidence intervals; *β*, correlation coefficients

Moreover, we have changed the word “correlation” to “relationship” throughout the new version of our revised manuscript because "relationship" suited better for results analyzing by linear regression analysis. "Correlation", on the other hand, is generally used for correlation analysis, which involves determining the degree of association between two continuous variables (*e.g.*, between an independent and a dependent variable or between two independent variables). Nevertheless, a regression analysis is a related technique to assess the relationship between an outcome variable and one or more risk factors or confounding variables. An outcome variable is also called a response variable or a dependent variable. Risk factors and confounders are called predictors, or explanatory or independent variables.

To avoid any confusion about correlation analysis, we substituted the word "correlated with" with "related to". The *β* values, which were added in Table 1 in the revised manuscript, are correlation coefficients involve multiplying the standard deviation (SD) by the ration of the dependent variable and the SD of the independent variable. The *β* parameters range from + 1 to -1. A positive *β* coefficient indicates a positive linear relationship between the independent and the dependent variable, whreas a negative *β* coefficient shows a negative linear relationship. Moreover, to the manuscript, we added an 95% confidence interval (CI), which is a range of values that is likely to contain the true population parameter value. A confidence level of 95 reflects a significant level of 5%.

In addition, we found that *P* values of histologic differentiation, TNM staging, T classification were different from the data presented the previous version as we previously used *P* values of the linear regression model, therefore, the *P* values of indicated the reliability of the overall independent variable (X) to predict outcome (Y). On the other hand, in this version of our revised manuscript, we re-analyzed the data using a *P* value of the independent variable and outcome, to show a statistically significant relationship between both independent variable and outcome. The results showed that there is a significant positive relationship between the XB130 expression with each of the following variables: stage III and stage IV of TNM staging, T3 and T4 classification.

To summarize, in this new version of our revised manuscript, we made some changes to our recent manuscript, including the Abstract, Materials and Methods, Results, and Discussion Sections as described as follows:

i.In the Abstract, on page 2, Line 30, the word “correlation” has been replaced by “relationship”;
ii.On page 2, Line 35-37, the sentence “Moreover, elevated XB130 expression levels were significantly correlated with lymphovascular space invasion (LVSI), intrahepatic type of CCA, stage IV of TNM staging, T4 classification and lymph node metastasis.” has been rewritten to “Moreover, elevated XB130 expression levels were positively related to lymphovascular space invasion (LVSI), intrahepatic type of CCA, high TNM staging (stage III, IV), high T classification (T3, T4), and lymph node metastasis.
iii.In the Introduction Section, on page 5, Line 109, the word “correlation” has been replaced by “relationship”;
iv.In the Method Section, on page 10, Line 222, the word “correlation” has been replaced by “relationship”;
v.In the Result Section, on page 13, Line 290, in the Results Section: the word “Correlation" has been replaced by “Relationship”;
vi.On page 13, Line 292, the word “correlation” has been replaced by “relationship”;
vii.On page 13, Line 294-302, the sentence “No correlation was observed between XB130 expression and the following variables: age (*P* = 0.976), gender (*P* = 0.958), histological type (*P* = 0.749), margin status (*P* = 0.110), histologic differentiation (*P* = 0.072) or M classification (*P* = 0.365). The XB130 expression was significantly correlated with LVSI (*P* = 0.005), intrahepatic type of CCA (*P* = 0.033), stage IV of TNM staging (*P* < 0.001), T4 classification (*P* < 0.001) and lymph node metastasis (*P* = 0.011)” has been rewritten to “No relationship was observed between XB130 expression and the following variables: age (*β* = -0.0023*, P* = 0.976), gender (*β* = 0.0042*, P* = 0.958), histological type (*β* = 0.0262, *P* = 0.749), margin status (*β* = -0.1305*, P* = 0.110), moderate differentiation (*β* = 0.1238*, P* = 0.128), poor differentiation (*β* = 0.1477*, P* = 0.070) or M classification (*β* = 0.074*, P* = 0.365). The XB130 expression was significantly positively related to LVSI (*β* = 0.2274*, P* = 0.005), intrahepatic type of CCA (*β* = 0.1623*, P* = 0.046), stage III of TNM staging (*β* = 0.4048*, P* < 0.001), stage IV of TNM staging (*β* = 0.2363*, P* = 0.012), T3 classification (*β* = 0.4314*, P* < 0.001), T4 classification (*β* = 0.3327*, P* < 0.001) and lymph node metastasis (*β* = 0.2060*, P* = 0.011)”;
viii.On page 13-15, our data in Table 1 have been edited and marked in red; and
ix.In the Discussion Section, on page 20, Line 394-397, the sentence “In CCA tissues, elevated level of XB130 expression by IHC was associated with LVSI, intrahepatic type of CCA, stage IV of TNM staging, T4 classification, and N1 classification (lymph node metastasis)” has been rewritten to “In CCA tissues, the elevated XB130 expression levels was positively related to lymphovascular space invasion (LVSI), intrahepatic type of CCA, high TNM staging (stage III, IV), high T classification (T3, T4), and N1 classification (lymph node metastasis)”
x.On page 21-22, Line 416-438, the Supporting information Section has been revised by adding the sentence

S1 Fig. Western blot analysis of XB130 expression in cell lines.

(PDF)

S2 Fig. Western blot analysis of β-actin expression in cell lines.

(PDF)

S3 Fig. Western blot analysis of phosphorylation of Akt expression.

(PDF)

S4 Fig. Western blot analysis of Akt expression.

(PDF)

S5 Fig. Western blot analysis of E-cadherin expression.

(PDF)

S6 Fig. Western blot analysis of vimentin expression.

(PDF)

S7 Fig. Western blot analysis of β-actin in media, scramble and siXB130.

(PDF)

S8 Fig. The Kaplan-Meier analysis.

(PDF)

S1 Data. Definition of TNM staging system.

(PDF)

S2 Data. Determination of protein expression by ImageJ.

(PDF)

S3 Data. Letter of consent.

(PDF)
